# Achieving high-performance room-temperature organic ferromagnetic semiconductor films via topochemical reduction

**DOI:** 10.1038/s41467-026-71866-2

**Published:** 2026-04-15

**Authors:** Yanuo Zhu, Qinglin Jiang, Hanlin Gan, Jiaji Yang, Xiandong He, Wei Cui, Shaohua Tong, Liang Yao, Jiang Zhang, Yuguang Ma

**Affiliations:** 1 Institute of Polymer Optoelectronic Materials and Devices, Guangdong Basic Research Center of Excellence for Energy and Information Polymer Materials, State Key Laboratory of Luminescent Materials and Devices, South China University of Technology, Guangzhou, China; 2 Guangdong Provincial Key Laboratory of Luminescence from Molecular Aggregates (South China University of Technology), Guangzhou, China; 3 Department of Physics, South China University of Technology, Guangzhou, China

**Keywords:** Organic molecules in materials science, Magnetic properties and materials, Electronic materials

## Abstract

The development of high-performance organic ferromagnetic semiconductors has been hampered by the intrinsic coupling of radical formation and structural organization during synthesis, which makes it difficult to achieve long-range magnetic coupling in highly conjugated systems. Here, we report an effective topochemical reduction strategy that decouples radical formation from structural organization, enabling unprecedented control over intermolecular arrangements in organic ferromagnetic materials. Using perylene diimide as a model system, this approach preserves the highly ordered structure of thermally evaporated precursor films during reduction, resulting in a shortened π-π stacking distance of 3.26 Å and exceptional long-range molecular order. The resulting films exhibit remarkable room-temperature ferromagnetism, as evidenced by X-ray magnetic circular dichroism, with a saturation magnetization of 10.5 emu g⁻^1^—nearly an order of magnitude higher than conventional organic magnetic materials—while retaining semiconducting properties. Generality of this strategy has also been demonstrated in naphthalene-based systems, underscoring its broad applicability. Theoretical calculations reveal that this enhanced performance originates from optimized ferromagnetic coupling between adjacent radicals through controlled twisted stacking configurations. This work provides a practical route to high-performance ferromagnetic semiconductors.

## Abstract (web summary)

Developing high-performance organic ferromagnetic semiconductors is hindered by the coupling between radical generation and molecular assembly during synthesis. Here, the authors use topochemical reduction to enable radical generation in pre-ordered perylene diimide films, maintaining their long-range order and achieving room temperature ferromagnetism and semiconducting behaviour

## Introduction

Ferromagnetic semiconductors possess the potential to bridge the fields of spintronics and conventional electronics by leveraging dual control over electron charge and spin, thereby transcending the limitations imposed by Moore’s law. These materials are anticipated to facilitate next-generation devices characterized by enhanced energy efficiency, data storage, and processing capabilities. However, traditional dilute magnetic semiconductors, which are typically fabricated by doping magnetic ions (e.g., Mn) into GaAs or InAs, encounter intrinsic limitations such as low Curie temperatures (*T*_c_) and inadequate saturation magnetization (*M*_s_)^1,2^. Moreover, despite significant advancements, two-dimensional transition metal-based ferromagnetic semiconductors still face challenges in achieving scalable synthesis and maintaining thermal stability at room temperature^3,4^.

Organic materials, distinguished by their lightweight and solution-processable nature with tunable electronic properties, provide a promising alternative to address these challenges. As early as 1963, McConnell hypothesized ferromagnetic coupling in organic π-radicals, sparking decades of exploration into organic magnetic systems^5^. Significant progress has been made in systems such as nitroxide radicals^6^ and graphene^7^; however, most reported materials exhibit *T*_c_ far below room temperature or negligible *M*_s_, thereby limiting their practical applications. Recent advancements in organic spintronics have demonstrated that delocalized π-radicals, when organized in highly ordered assemblies, can substantially enhance magnetic coupling and thermal stability^8,9^. Nevertheless, the simultaneous achievement of high saturation magnetization (*M*_s_ > 1 emu g^-1^), room-temperature Curie temperature (*T*_c_ > 300 K), and semiconducting properties in purely organic systems remains an elusive goal.

In organic ferromagnets, the strength of intermolecular radical interactions dictates the Curie temperature, while the number of molecules forming ferromagnetically coupled packing structures determines the saturation magnetization^10^. For radicals with limited conjugation, such as *p*-NPNN^11^, the weak intermolecular coupling facilitates molecular assembly and even single-crystal growth, resulting in high saturation magnetization only at low temperatures. In contrast, radicals with extended conjugation, such as perylene diimide (PDI) aggregates^12^, exhibit strong intermolecular interactions but are challenging to assemble in a controlled manner (Supplementary Fig. 1). Although their Curie temperatures can approach or exceed room temperature, their saturation magnetization remains far below the theoretical value. This highlights a fundamental bottleneck in achieving high-performance organic ferromagnetic semiconductors: establishing strong ferromagnetic coupling across all radicals in large conjugated systems. This bottleneck stems from a central conflict in most synthetic strategies: radical generation is inherently coupled with the assembly process. The coupling often leads to undesired aggregate structures that favor non-ferromagnetic interactions, undermining the targeted magnetic ordering.

To address the challenge, we introduce topochemical reduction—a strategy involving in-situ chemical conversion on pre-ordered molecular films—that can decouple the generation of radicals from the construction of aggregate structures as a universal approach for fabricating high-performance organic ferromagnetic semiconductor films^13^. By performing chemical reduction on pre-ordered molecular films rather than solution process, this approach enables in-situ radical generation within highly ordered molecular assemblies while preserving precise intermolecular arrangements. Utilizing PDI as a model system, our topochemically reduced films achieve an unprecedented combination of robust room-temperature ferromagnetism, high saturation magnetization and semiconducting properties with reduced π-π stacking distances (3.26 Å) that significantly enhance magnetic coupling. The universality of this approach is validated through successful extension to naphthalene diimide systems. These results provide a universal method for preparing high-performance organic ferromagnetic semiconductor films.

## Results

### Topochemical reduction of PDI films

**Fig. 1:**
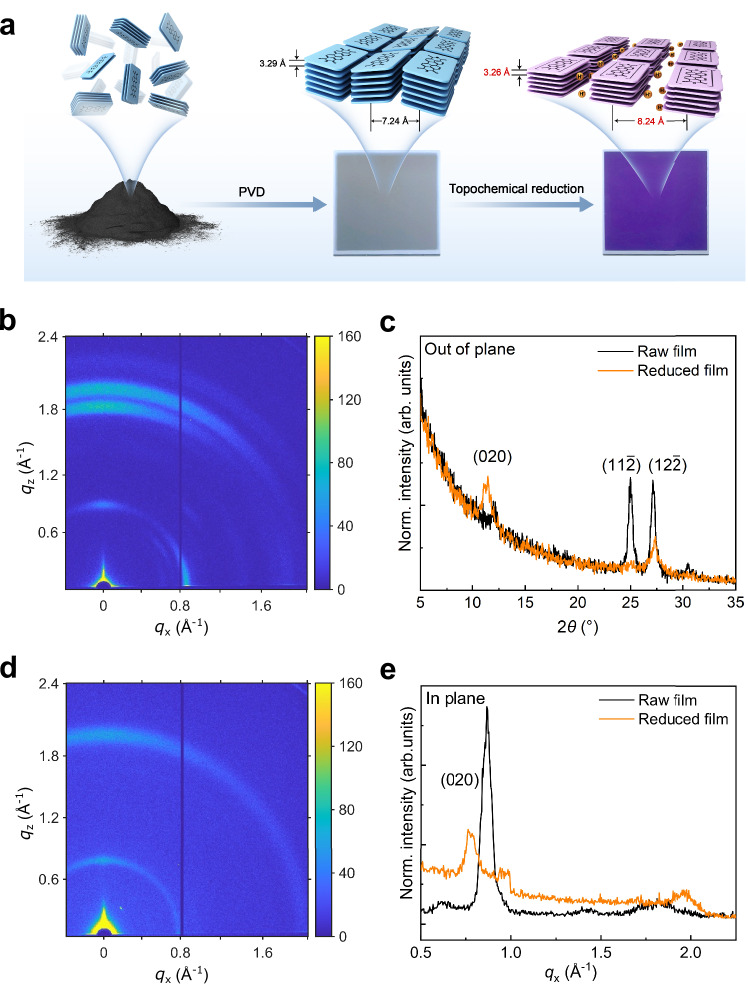
Topochemical reduction of PDI films. **a** Schematic diagram of preparation of ferromagnetic semiconductor films. An ordered structure was formed through thermal evaporation from polycrystalline powder of PDI, and the arrangement was preserved after a topochemical reduction. **b** GIWAXS pattern of raw films. Raw films exhibit face-on orientation. **c** Comparison of GI-XRD pattern between raw films and topochemical-reduced films. The crystallinity decreased, and the peak at 27.12°in raw films moved to 27.35° in topochemical-reduced films, implying a closer π-π distance. **d** GIWAXS pattern of reduced films. **e** Comparison of in-plane integral image extracted from GIWAXS patterns. The peak at 0.87 Å^-1^ in raw films moved to 0.76 Å^-1^ in reduced films, implying the insertion of protons.

Topochemical synthesis can be broadly defined as adding, extracting or substituting elements to or from precursors in an environment for synthesis of new materials which retain the structure. Here, this strategy enables the reduction of PDI within ordered films, decoupling radical generation from structural ordering. Figure 1a demonstrates the preparation schematic diagram of the ferromagnetic semiconductor films. The preparation of ferromagnetic semiconductor films begins with thermally evaporated PDI films (Supplementary Fig. 2a, b), which exhibit an ordered stacking structure. Following topochemical reduction using hydrazine hydrate vapor (Supplementary Fig. 2c, d), the molecular arrangement undergoes a transformation into a flattened and reduced configuration. The structure of the films was analyzed using grazing incidence wide-angle X-ray scattering (GIWAXS) and grazing incidence X-ray diffraction (GI-XRD). Figure 1b shows the GIWAXS patterns of raw films. Raw films exhibited good orientation, with diffraction rings in the range of 1.8–2.0 Å^−1^ showing significantly higher intensity in the z-axis direction than x-axis direction, combining data from powder diffraction (Supplementary Fig. 3), indicating that PDI molecules were stacked along the z-axis direction and had a face-on orientation. At the same time, diffraction rings near 0.9 Å^−1^ had higher intensity in the x-axis direction, indicating that the lamellar packing had an edge-on orientation.

By capturing the GI-XRD along the out-of-plane direction (Fig. 1c), we can see that the raw films present sharp diffraction peaks, indicating that the films had good crystallinity. The peak at 27.1° stands for the crystal plane (12$\bar{2}$) with an interplanar spacing (π-π distance) of 3.29 Å, while the peak at 25.1° points out the crystal plane (11$\bar{2}$), meaning a zigzag π-π stacking plane, and the peak at 12.0° represents the crystal plane (020) or lamellar packing.

According to McConnell’s theory, a high concentration of paramagnetic centers (or radicals) is a prerequisite for obtaining ferromagnetism in organic systems. Therefore, we tracked the electron paramagnetic resonance (EPR) spectra of PDI films with different topochemical reduction duration to study the content of radicals (Supplementary Fig. 4a). Raw film was EPR silent, and as the reducing duration increased, the first-order differential EPR signal gradually intensified until reaching its peak at 6 h, The EPR signal exhibited an asymmetric peak centered at *g* = 2.003 (Supplementary Fig. 4b), consistent with the Landé factor of electrons and indicating radical formation. At the same time, the delocalization of electrons led to the asymmetry of the peak shape^14,15^. With extended reduction, the EPR signal weakened due to further reduction of PDI radical anions to EPR-silent dianions. We performed quadratic integration on the first-order differential signal and obtained the curve of the relative content of radicals as a function of processing duration (Supplementary Fig. 4c). We further confirmed the valence state of PDI in the film through UV spectra (Supplementary Fig. 4d). The raw films exhibited absorption characteristic of neutral PDI aggregates, with peaks at 479, 509, and 573 nm. After 24 h of topochemical reduction, a central absorption peak appears at 551 nm, which is consistent with typical PDI dianion absorption reported in previous literature^16^. The films treated with saturated steam of hydrazine hydrate solution for 6 h show an absorption spectrum between the two above, with weak absorption appeared at 730, 816, and 976 nm, which were within the wavelength range of absorption of PDI radical anions reported in the literature^17,18^. We attempted to perform a quantitative analysis using X-ray Photoelectron Spectroscopy (XPS), but the results were not reliable (Supplementary fig. 4e, f). Ultimately, we use EPR data to validated that 6 h represented optimal timing for obtaining films with maximum radical content under this process.

After 6 hours of topochemical reduction, the RMS roughness of the original flat raw films increased from 2.44 nm to 4.83 nm (Supplementary Fig. 5). The crystallinity and orientation of the film (extract from peak intensity in GI-XRD and azimuth integration in GIWAXS) were reduced (Fig. 1d and Supplementary Table 1). The diffraction peak corresponding to the (11$\bar{2}$) plane disappears, the (020) peak shifts to lower angles and no additional diffraction peaks emerge after reduction, suggesting that the films do not undergo a complete structural reconstruction into a new crystalline phase. The π-π distance of molecules figured out from the shift of (12$\bar{2}$) peak reduced to 3.26 Å, which was significantly different from the π-π spacing of neutral PDI. This may due to the interaction between additional radicals. The changes of lamellar packing distance read from the integral image of GIWAXS (Fig. 1e) was determined to increase by 1 Å, possibly due to the insertion of counter cations (speculated to be protons, considering the diameters of other cations) generated during the reduction, causing lattice expansion. It is worth noting that compared with previous reports in which radical generation is accompanied by uncontrollable aggregation during solution-phase evaporation, topochemical reduction can decouple radical generation from structural assembly, pre-build the PDI molecules in the film in a well-organized state and obtain a high concentration of radicals in an orderly stacked ionic PDI film. Along with the shorter π-π distance of 3.26 Å, the films are expected to exhibit superior performance.

### Magnetic properties

**Fig. 2:**
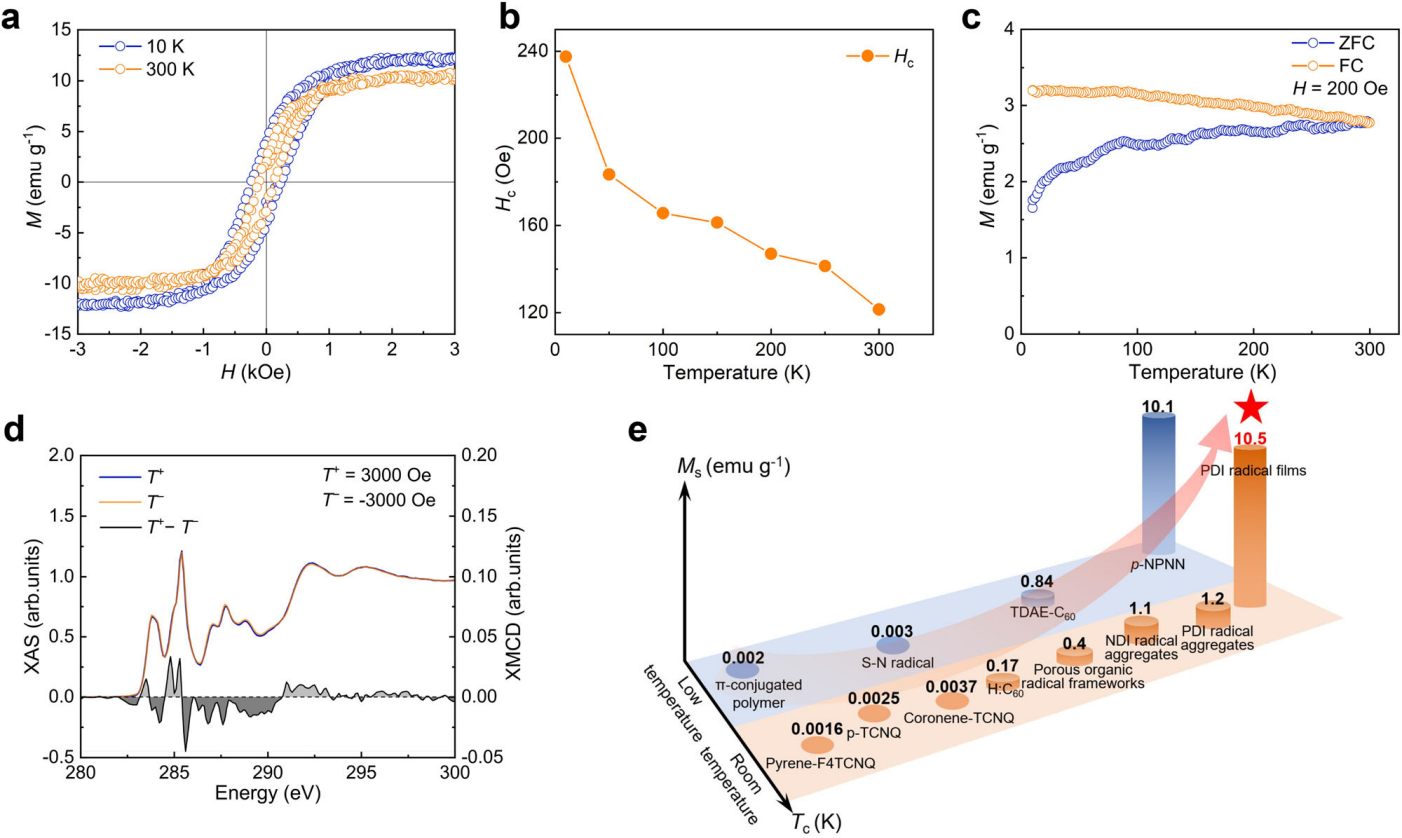
Magnetic properties of the films. **a** *M*-*H* hysteresis loops of the films taken at 300 K and 10 K. **b** Temperature dependence of the coercive fields, and suggesting ferromagnetism at room temperature. **c** Curves of zero-field-cooled (ZFC) and field-cooled (FC) magnetization with temperature, measured at an applied magnetic field of 200 Oe. The bifurcation between the curves implies that the *T*_c_ of the films was beyond 300 K. **d** XAS (blue and orange line) and XMCD (black line, light and dark grey area) spectra of the films. The peaks in the range of 283–290 eV indicate that the origin of ferromagnetism is π electrons in perylene. **e** Comparison of *M*_s_ and *T*_c_ in purely organic magnets. Detailed properties can be seen in Supplementary Table 4.

Physical Properties Measurement System (PPMS) was used to investigate the magnetic properties of the films. The *M*-*H* curves measured at temperatures of 10 K and 300 K exhibited a distinct hysteresis loop of ferromagnetic materials (Fig. 2a), with magnetization saturating at approximately 1 kOe and a coercivity of 121.5 Oe at 300 K. We determined the mass of PDI on the film to be 5.95 μg by measuring the absorption of the solution (Supplementary Fig. 6). After subtracting the background signal of the silicon wafer, we calculated the *M*_s_ of the film to be 10.5 emu g^−1^, nearly ten times higher than that observed in the powder samples and 73.4% of theoretical maximum (1 *μ*_B_ per molecule). Furthermore, we measured the hysteresis loop in the temperature range of 10-300 K and found that the coercivity increased with the decreasing temperature (Fig. 2b and Supplementary Fig. 7), which was caused by the gradual freezing of spin. Under an external magnetic field of 200 Oe, the changes in magnetic susceptibility with temperature during zero field cooling and field cooling processes are shown in Fig. 2c. The two curves coincide at 300 K, indicating that the *T*_c_ of the ferromagnetic film was above 300 K. We also measured the films reduced for different times (Supplementary Fig. 8). Those for 2 h, 12 h, and 48 h exhibit paramagnetic behavior in the measured field range, whereas the 4 h film shows a weak approach to saturation above ~2000 Oe with an almost negligible coercivity, consistent with the onset of extremely weak ferromagnetic coupling when a substantial radical population begins to form but before the coupling becomes robust indicating that strong ferromagnetism emerges only when the radical population and the coupling efficiency become sufficiently high.

In order to determine the source of ferromagnetism, we conducted X-ray magnetic circular dichroism (XMCD) testing. XMCD is a direct method for identifying the elemental source of magnetism. The optical spiral vector arranged parallel (or antiparallel) to the orbital moment will excite electrons with spin up (or spin down) to available spots in the unoccupied valence band, resulting in a net positive or negative peak in the XMCD spectrum, with peak intensity depending on the relative number of spin up and spin down holes^19,20^. The C K-edge XMCD measured at room temperature with light incident perpendicular to the film plane and an applied magnetic field of 3000 Oe is shown in Fig. 2d. The absorption at 283–290 eV comes from the π^*^ resonance of the perylene nucleus, while the peak in the range of 290–300 eV is attributed to σ^*^ resonance. The peak at 284 eV originates from the transitions of the electrons in the perylene core from C1*s* to lowest Unoccupied Molecular Orbital (LUMO). The peak at 285.5 eV can be classified as a transition from core levels to orbitals with higher energy. The absorption in the range of 287–288 eV can be associated with transitions of electrons on the imide moiety from core level to LUMO. Subsequently, the XMCD curve is obtained by calculating the difference between the normalized X-ray absorption spectroscopy (XAS) spectra measured in opposite magnetic fields. Given the condition that the maximum XAS and XMCD were 1.21 and 0.0785, respectively, the strength of XMCD reached 6.5%, which was on the same order of magnitude as other metal-organic ferromagnetic semiconductors reported in PDI derivative and Co(Cp)_2_/SnS_2_^21,22^. The absorption above 290 eV, which represents transitions of σ electrons, exhibits a broader peak without unmistakable XMCD signal. The XMCD signal up to 6.5% confirms the ferromagnetism from the polarized π electrons in the PDI films.

To eliminate the influence of metal impurities on magnetic testing, we conducted ICP analysis on sublimated neutral PDI powders and topochemical-reduced PDI films (Supplementary Table 2, 3). The ferromagnetic impurities in the film can only provide an *M*_s_ of no more than 3.3 × 10^-3^ emu g^−1^, far less than the values we measured. The magnetic properties of raw films and those stored in N_2_ glove box for 5 months and in air for 30 days are shown in Supplementary Fig. 9a-c. Raw films are diamagnetic due to its close-shell characterization. Films placed in N_2_ show weaker ferromagnetism, while after 30 days in air exhibiting diamagnetism. Supplementary Fig. 9d shows the diamagnetic *M*-*H* curve of ferromagnetic film after thermal decomposition, which can also rule out the existence of ferromagnetic impurity.

Figure 2e illustrates the reported values of *M*_s_ and *T*_c_ for purely organic room-temperature ferromagnets. A majority of these materials either exhibit *T*_c_ well below ambient conditions or possess very low *M*_s_, rendering the simultaneous attainment of both high *M*_s_ and elevated *T*_c_ a significant challenge. Previous methods for synthesizing organic ferromagnets have often failed to facilitate robust interactions and ordered arrangements among radicals. By utilizing the topochemical reduction approach, this limitation is effectively addressed, thereby enabling the achievement of high *M*_s_ and room-temperature ferromagnetism in PDI radical films.

### Electrical Properties

We utilized home-made equipment (Supplementary Fig. 10a, b) to continuously monitor the changes in the conductivity of the film in real-time during the reduction. Upon exposure to saturated steam, the initially neutral PDI underwent rapid reduction, leading to the formation of radical anions and dianions within the film, thereby transforming it into a doped state, resulting in alterations in its conductivity behavior (Supplementary Fig. 10c), and the conductivity at 6 h was 0.90 S cm^−1^.

**Fig. 3:**
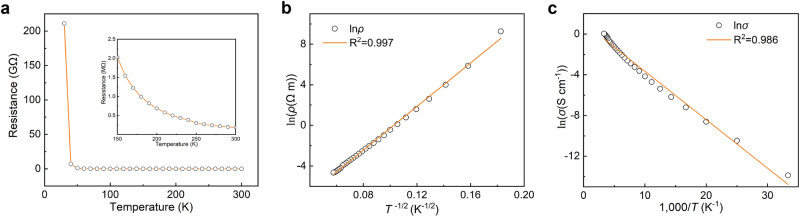
Electrical properties of reduced films. **a** Film resistance at different temperature. As the temperature decreases, the resistance increases exponentially, which is a typical characteristic of semiconductors. **b** The linear relationship between natural logarithm of resistivity (ln*ρ*) and *T*^-1/2^, conforming to 1D-VRH conduction model. **c** The relationship between natural logarithm of conductivity (ln*σ*) and *T*^-1^, and the result of linear fitting gives an apparent activating energy of 41 meV.

In order to investigate its semiconductor properties, we used four-point probe method on LakeShore 8400 system to test the variable temperature resistance of the film (Supplementary Fig. 10d). At 300 K, the conductivity was 1.05 S cm^−1^, close to the data obtained using our self-made equipment. After the temperature dropped to 30 K, the conductivity decreased to 9.47 × 10^-7^ S cm^−1^. During the cooling process of 300–30 K, the resistance of the film increases exponentially, which once again determines the semiconductor properties of the film (Fig. 3a). The data were fitted using the 1D-VRH, 2D-VRH, 3D-VRH, and Arrhenius models. The linear dependence of *T*^−1/2^ and natural logarithm of resistivity (ln *ρ*) fits the best, confirming the 1D-VRH conduction model in the film (Fig. 3b)^23,24^. The relationship between reciprocal temperature (*T*^−1^) and natural logarithm of electrical conductivity (ln *σ*), as described by the Arrhenius equation, gives a typical thermal excitation model for semiconductors with an apparent activation energy value at approximately 41 meV (Fig. 3c)^25^.

We validated the universality of the method by employing naphthalene diimide (NDI, Supplementary Fig. 11a), a material extensively utilized in organic electronics for its conjugated π-electron system, to fabricate radical films. The raw films exhibited a sharp peak at 28.1° (Supplementary Fig. 11b), representing a (11$\bar{2}$) interplane distance (Supplementary Fig. 11c) or π-π distance of 3.17 Å, and the molecules had a face-on orientation (Supplementary Fig. 11d). After topochemical reduction, the relative radical concentration reached a peak (Supplementary Fig. 12a), exhibiting characteristic spectrum of NDI radical anions (Supplementary Fig. 12b). X-ray scattering and diffraction analysis (Supplementary Fig. 13) reveals a reduction in film crystallinity and an increase in π-π stacking distance to 3.26 Å. After eliminating the interference of metal impurities (Supplementary Table 5), the NDI radical films presented a room-temperature *M*_s_ of 10.0 emu g^−1^ (Supplementary Fig. 14) along with semiconductivity (Supplementary Fig. 15).

### Theoretical calculation

**Fig. 4:**
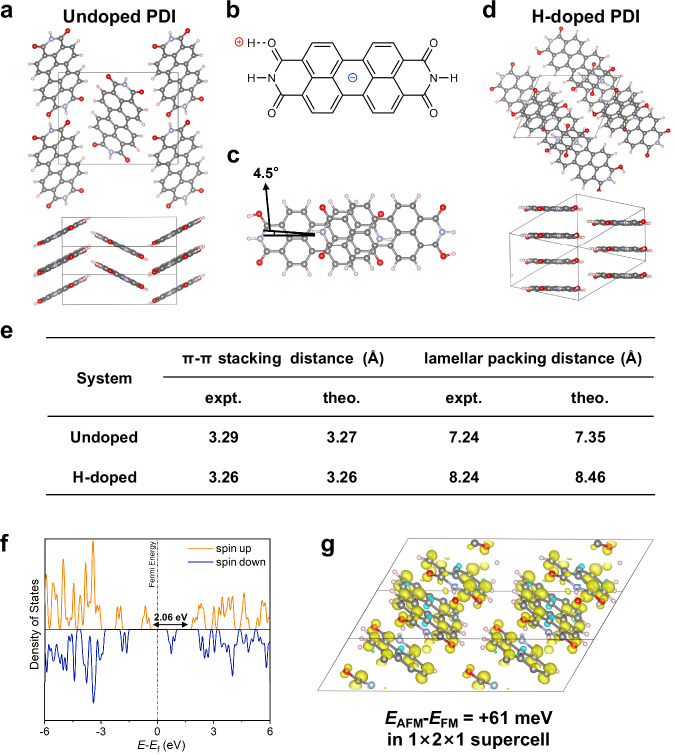
Ferromagnetic coupling of the H-doped PDI film. **a** Two views of theoretically optimized unit cell structure of undoped PDI. **b** Chemical structure of H-doped PDI radical unit. **c** Twisted stacking structure between the adjacent radical units within the unit cell of H-doped PDI. **d** Two views of the theoretically optimized unit cell structure of H-doped PDI. **e** Comparison of computational and experimental data. **f** Density of states (DOS) for H-doped PDI unit cell. **g** Spin density representation (isovalue = 0.003 a. u.) and energy difference between antiferromagnetic (AFM) and ferromagnetic (FM) states of H-doped PDI 1×2×1 supercell.

To elucidate the relation between ferromagnetism and aggregation structure of PDI anion radicals, the first-principle calculations were conducted for the periodic models of pristine and topochemical-reduced PDI films. Firstly, the calculation on the pristine PDI single crystal (Fig. 4a) was performed to validate the accuracy of the theoretical methods. The computational π-π stacking and lamellar packing distance of pristine PDI are 3.27 Å and 7.35 Å respectively, which agrees well with the experiment. It is indicated that the PBE-D3(BJ) functional is appropriate for describing the condensed phase of PDI systems. The radical unit in the ferromagnetic film is PDI anion radical, with proton acting as counterion. The n-type doped PDI (H-doped PDI) was modeled by adding an electron and a proton to a C = O group (Fig. 4b). Based on GIWAXS and GI-XRD data, a possible periodic model (containing two hydrogen-doped PDI units) for H-doped PDI film was constructed. Subsequently the atomic positions and lattice constants were optimized at the same theoretical level as pristine PDI. Computational results indicate that H-doping leads to a 4.5° twisted stacking structure between adjacent radicals (Fig. 4c and Supplementary Table 6) and a transformation of crystal structure from herringbone to sheet-like packing (Fig. 4d). The lamellar packing distance is increased to 8.46 Å. In addition, the calculated π-π stacking distance (3.26 Å) shows a slight shortening due to H-doping. These theoretical results regarding crystal packing are consistent with the experimental observations of the topochemical-reduced PDI films (Fig. 4e).

We performed electronic structure calculations on the optimized H-doped PDI primitive cell at the PBE0-D3(BJ) level of theory to reduce the inherent self-interaction error in PBE-D3(BJ) functional^26^, which can result in an underestimation of the bandgap. The spin-resolved atom-projected DOS (PDOS) in Supplementary Fig. 16 shows that the states in the vicinity of the Fermi level are dominated by contributions from C atoms in the perylene π-core. The O atoms in the imide moiety exhibit only minor weight near the Fermi level, while the contribution from N atoms is essentially negligible. These results indicate that the spin polarization predominantly resides on the carbon-based π-framework, supporting for the experimental XMCD measurements. The density of states (DOS) revealed the characteristics of a typical ferromagnetic semiconductor (Fig. 4f). The bandgap for the *α* spin channel is approximately 2.06 eV, falling within the range observed for common semiconductors. It is worth to mention that the experimental excitation energy of the topochemical-reduced PDI film was 2.16 eV (Supplementary Fig. 4 d). Additionally, there is significant exchange splitting near the Fermi level: the upper edge of the *α* spin DOS lies higher in energy than the upper edge of the *β* spin DOS, indicative of ferromagnetic behavior. The calculated magnetic moment is 2.00 *μ*_B_ per cell.

In order to understand the spin coupling within and between the radical stacks, a 1×2×1 supercell model was constructed from the aforementioned H-doped PDI primitive cell to calculate the energy difference between the ferromagnetic (FM) and antiferromagnetic (AFM) states at PBE0-D3(BJ) level. The results indicate that the H-doped PDI supercell exhibits three-dimensional ferromagnetism, with the AFM state being 61 meV higher in energy than the FM state (Fig. 4g). This energy difference surpasses Landauer limit^27–29^ at room temperature (*k*_B_*T*·ln2 = 18 meV), suggesting that the *T*_c_ of the ferromagnetic semiconducting material exceeds room temperature. The ferromagnetic coupling between H-doping PDI units within the stacks is likely attributed to the twisted and slipped stacking configuration, which results in minimal SOMO-SOMO overlap. Furthermore, the ferromagnetic coupling between the stacks is likely a result of their tight packing.

## Discussion

In this work, we overcome these challenges faced by organic ferromagnetic semiconductors through topochemical reduction, successfully fabricating highly ordered PDI radical films. These films demonstrate unprecedented magnetic properties, including a remarkable *M*_s_ of 10.5 emu g^−1^ and above-room-temperature *T*_c_, representing a tenfold improvement over existing PDI aggregates. Furthermore, the films exhibit semiconductor behavior, highlighting their potential for organic spintronic applications.

Based on our experimental findings, we systematically investigated the design principles of organic ferromagnetic semiconductors. Firstly, at the molecular level, stable radicals serve as the fundamental building blocks for constructing room-temperature ferromagnetic semiconductors. While conventional nitroxide and triphenylmethyl radicals demonstrate high kinetic stability, their localized electronic structures inherently limit intermolecular magnetic interactions. In contrast, organic radicals featuring delocalized π-electron structures offer dual advantages: enhanced stability through delocalization effects, which reduces recombination probability, and stronger intermolecular interactions via π-orbital overlap. Consequently, extensively conjugated molecules such as PDI and NDI (Supplementary Fig. 17a, b) emerge as ideal candidates for researches^30^.

Secondly, and most importantly, at the aggregate structural level, we revealed the decisive influence of molecular packing configurations on magnetic properties. Structural parameters such as π-π distance, slip length, and twist angle directly affect the magnitude of spin interactions between adjacent radicals, thereby modulating both the microscopic magnetic coupling modes and the macroscopic *T*_c_. In systems with weak spin interactions, such as localized radical aggregates (Supplementary Fig. 17c), the limited orbital overlap results in relatively isolated spins, leading only to paramagnetism or weak ferromagnetism at low temperatures^31–33^. Conversely, when spin interactions become too strong, which is common in delocalized radicals, adjacent molecules tend to form multi-center two-electron bonds (pancake bonding), resulting in a ground state that favors antiferromagnetic coupling^34,35^. Notably, increasing the intermolecular twist angle to maintain a moderate spin interaction can induce a ferromagnetic coupling state (Supplementary Fig. 17d) that energetically supersedes the Pancake Bonding configuration (Supplementary Fig. 17e) and the *T*_c_ increased with system size^36^. Our topochemically reduced films demonstrate superior long-range order compared to PDI radical powders, enabling more extensive radical participation in ferromagnetic coupling. This enhanced ordering appears crucial for the films’ exceptional magnetic performance. Additionally, electron delocalization confers semiconductor properties, resulting in multifunctional ferromagnetic semiconductor films.

This research not only experimentally realized PDI films exhibiting room-temperature ferromagnetism and semiconductor properties but also established a molecular design paradigm for organic ferromagnetic semiconductors from a theoretical perspective. Topochemical reduction involves selecting radicals with strong delocalization characteristics as basic units and achieving long-range ordered ferromagnetic coupling through precise control of molecular packing. This method produces organic ferromagnetic semiconductor films with *M*_s_ of 10.5 emu g^−1^ and *T*_c_ over 300 K, along with a semiconductor behavior. Our findings advance the practical applications of room-temperature organic ferromagnetic semiconductors, laying a solid foundation for innovative developments in organic spintronics.

## Methods

### Sample Preparation

Perylene diimide (PDI) (98%), naphthalene diimide (NDI) (95%) and dimethyl sulfoxide (DMSO) were purchased from J & K Co., Ltd. Sublimation technology was used to obtain pure PDI and NDI ( > 99%). Hydrazine hydrate (98% in water) was obtained from Shanghai Aladdin Biochemical Technology Co., Ltd (Shanghai, China). A high-resistance silicon wafer with a (100) orientation was subsequently cleaned with sonication in water, cleaning agent, acetone, and isopropanol. Then PDI was thermally evaporated onto the silicon wafer at a speed of 0.1 Å s^-1^ for the first 10 nm, and 0.3 Å s^-1^ for the rest 90 nm to obtain raw films. 50% Hydrazine hydrate (diluted by DMSO) was added into a homemade sealed bottle in an N_2_ glovebox (H_2_O & O_2_ < 1 ppm) to form saturated vapor of hydrazine hydrate, and then the raw film was placed inside for a certain time to obtain topochemical-reduced films.

### Component analysis

The UV-Vis spectra were recorded on an Ocean QE65000 spectrometer in an N_2_ glovebox (H_2_O & O_2_ < 1 ppm). EPR spectra were recorded on a Bruker E500 EPR spectrometer (300 K, 9.849 GHz, X-band, Karlsruhe, Germany). The microwave power used was 0.6325 mW and the width of the magnetic field sweep ranged from 3250 to 3700 Oe. The modulation frequency was 100 kHz and its amplitude was 5 Oe. ICP-MS analysis was conducted on PerkinElmer Optima 8300.

### Structure Characterization

The crystallinity of the samples was characterized using GI-XRD (Rigaku SmartLab) with Cu Ka (λ = 0.15418 nm) radiation and GIWAXS (Xenocs Xeuss 2.0) with liquid metal target (λ = 0.13414 nm) radiation. The atomic force microscopy (AFM) images were obtained from using a Innova atomic force microscope (SPM).C K-edge XAS and XMCD spectra of films were collected at beamline BL12B-a of the National Synchrotron Radiation Laboratory (NSRL), Hefei, and the signal of the total electron yield was measured at 300 K under a vacuum better than 10^-7^ Pa. XAS were recorded for 3 times under each magnetic-field direction (±3000 Oe) to ensure reproducibility, and the XMCD spectrum was obtained from the difference between the averaged XAS spectra measured under opposite fields. The XAS signals were processed using simple normalization as *I*/*I*_0_ where *I* is the total electron yield signal of films and *I*_0_ is the photocurrent from a gold mesh as a reference.

### Physical Properties Measurement

Magnetization of films with a size of 3×10 mm^2^ was measured using Quantum Design PPMS-9 with a vibrating sample magnetometer over the temperature range of 10-300 K. The diamagnetic correction was performed using diamagnetic susceptibility from the sample holder. Variable temperature resistance test was carried out on LakeShore 8400 over the temperature range of 30-300 K.

### Computational Details

Spin-polarized DFT calculations were performed using the plane-wave-pseudopotential scheme available in the Quantum Espresso^37–39^ package (version 7.0). PBE exchange correlation functional^40^ with Grimme’s dispersion correction of D3 version (Becke-Johnson damping)^41^ was employed for the calculations of ionic minimization and variable cell relaxation. The standard solid-state pseudopotentials (SSSP) library^42^ optimized for efficiency (SSSP PBE Efficiency v1.3.0) was generally employed. The corresponding valence electrons considered for the calculations were 2*s*^2^2*p*^2^, 2*s*^2^2*p*^3^, 2*s*^2^2*p*^4^ for C, N and O atoms, respectively. Cutoffs used in kinetic-energy and charge density were 60 Ry and 480 Ry respectively. The k-point mesh applied to sample the Brillouin zone was 4×2×2, which was generated with the Monkhorst-Pack scheme. The SCF energy convergence threshold was set to 1.0×10^-6 ^Ry, and the force convergence standard was set to 1.0×10^-4 ^Ry/Bohr. The internal stress convergence standard was set to 0.3 kbar, and convergence threshold on total energy for ionic minimization was set to 1.0×10^-4^ Ry. Afterwards, global hybrid functional PBE0^43^ was adopted to calculate the density of states of primitive cell and the energy difference between antiferromagnetic and ferromagnetic states of 1×2×1 supercell. The VESTA package^44^ was used to visualize the results on the periodic structures.

## Supplementary information

Supplementary Information

Transparent Peer Review file

## Source data

Source Data

## Data Availability

The raw experimental data in this study and Cartesian coordinates of all DFT-optimized structures are available in the Source Data file. All data are available from the corresponding author upon request. Source data are provided with this paper.
