# Supplementary material for: Achieving high-performance room-temperature organic ferromagnetic semiconductor films via topochemical reduction
Source: Nat Commun. 2026 Apr 15;17:5197. doi: 10.1038/s41467-026-71866-2 (PMC13253818; doi:10.1038/s41467-026-71866-2)
Supplement: Supplementary file 1 — Supplementary Information [file 41467_2026_71866_MOESM1_ESM.pdf]

Supplementary Information

Achieving High-Performance Room-Temperature Organic Ferromagnetic Semiconductor Films via Topochemical Reduction

Yanuo Zhu[1, 2], Qinglin Jiang[1, 2, *], Hanlin Gan[1, 2], Jiaji Yang[1, 2], Xiandong He[1, 2], Wei Cui[1, 2], Shaohua Tong[1, 2], Liang Yao[1, 2], Jiang Zhang[3, *], Yuguang Ma[1, 2, *]

[1]Institute of Polymer Optoelectronic Materials and Devices, Guangdong Basic Research Center of Excellence for Energy and Information Polymer Materials, State Key Laboratory of Luminescent Materials and Devices, South China University of Technology, Guangzhou 510640, China

[2]Guangdong Provincial Key Laboratory of Luminescence from Molecular Aggregates (South China University of Technology), Guangzhou 510640, China

[3]Department of Physics, South China University of Technology, Guangzhou 510640, China

*Corresponding emails: jiangql@scut.edu.cn; jonney@scut.edu.cn; ygma@scut.edu.cn

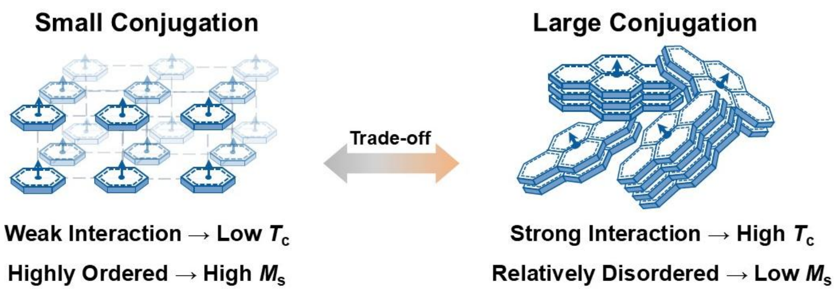

Supplementary Fig. 1 Conceptual trade-off between conjugation size, spin interaction, and achievable ferromagnetic performance in organic radical systems.

Neutral PDI was deposited on Si. We treated the film in a sealed Schott bottle containing hydrazine hydrate solution, and the treated films were obtained after certain hours.

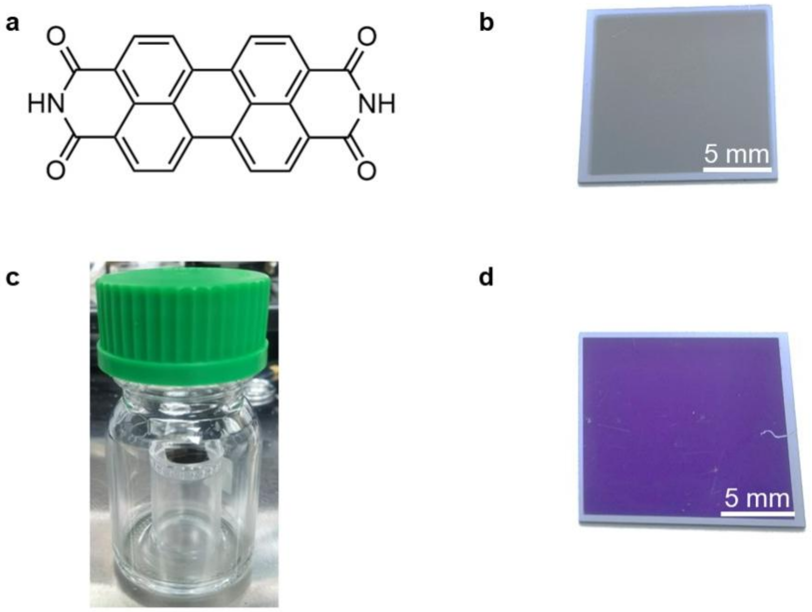

Supplementary Fig. 2 Digital photos of testing equipment and films. **a** Molecular formula of PDI. **b** Digital photo of raw film. **c** Homemade bottle used to reduce films. **d** Digital photo of the film after reduction.

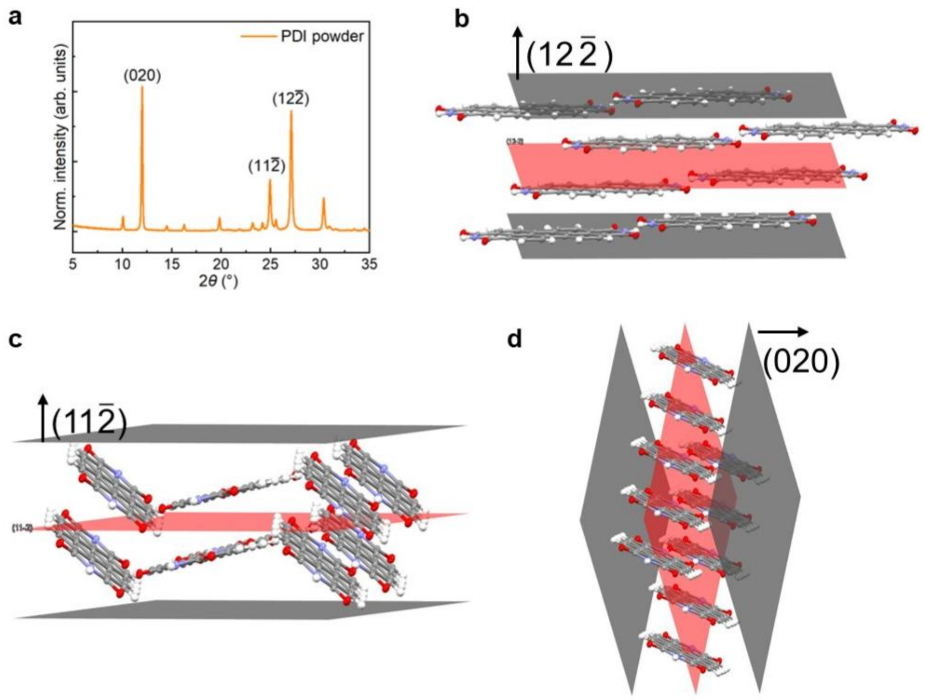

Supplementary Fig. 3 Structure of PDI powders. **a** Powder-XRD patterns of PDI. Molecular arrangements on crystal planes extracted from PDI single crystal and powder diffraction data are presented in **b** (12$\bar{2}$), **c** (11$\bar{2}$), **d** (020).

The signal intensity increased for the first 6h and then decreased. So it was chosen to be the best topochemical reducing time. UV spectra confirmed that the EPR signal came out from ionized PDI.

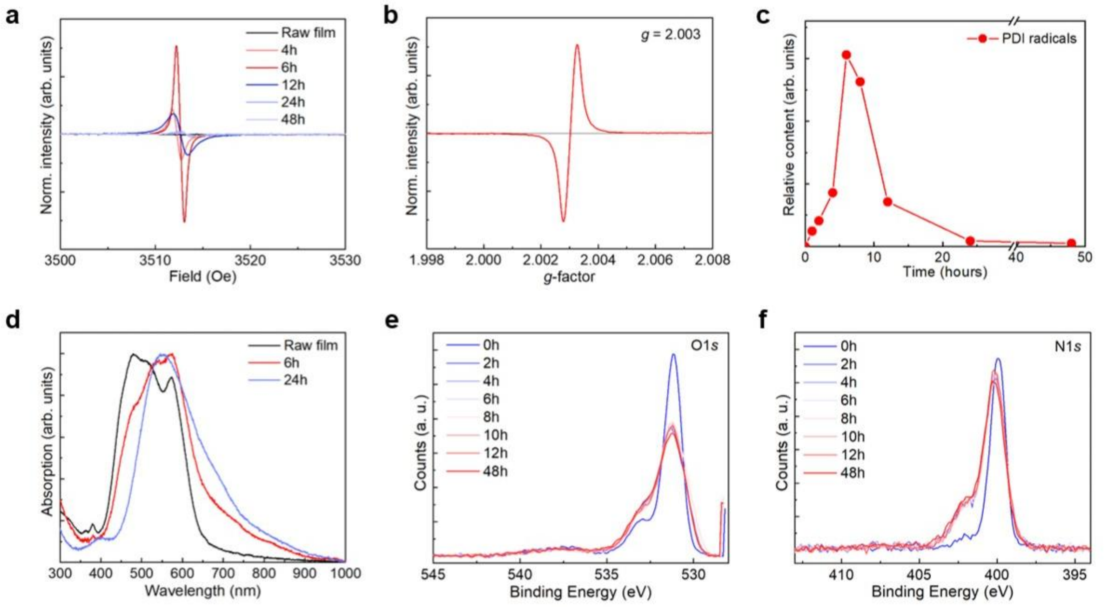

Supplementary Fig. 4 Film composition. **a** EPR spectra of raw films and films reduced for 4h, 6h, 12h, 24h, 48h. **b** *g*-factor of the electrons in the films. **c** Changes of relative content of radicals in the films over reducing time. **d** UV-vis spectra of raw films and films reduced for 6h, 24h. Binding energy of N1*s* (**e**) and O1*s* (**f**) electrons in films with different reduction time.

The roughness of topochemical-reduced films increased a little from 2.44 nm to 4.83 nm.

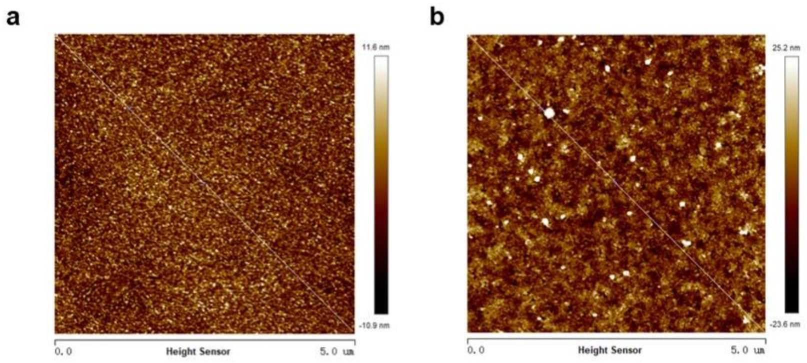

Supplementary Fig. 5 Surface morphology of films. **a** AFM image of raw films, showing a flat surface without obvious morphological features. **b** AFM image of topochemical-reduced films.

We determined film mass from solution absorbance.[1-5] A series of concentrations of PDI solutions were prepared. Considering the final concentrations below 10^{-4} mol/L, the PDI species were fully converted into dianion state. By fitting the standard curve, we calculated its slope to be 26.54 and intercept to be 1.6508×10^{-4}. After fully dissolving the film in hydrazine hydrate, the absorbance of the solution at 553 nm was 0.08097, which was incorporated into the curve to obtain a film weight of 5.95 μg.

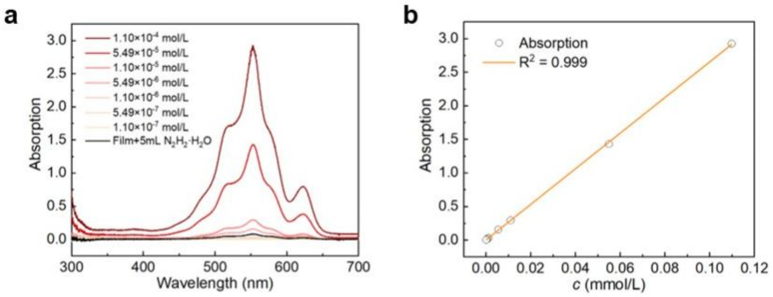

Supplementary Fig. 6 Determination of film weight. **a** UV-Vis spectra of hydrazine solution of PDI with different concentrations. **b** Standard curve of absorption-weight.

M_s and coercivity of topochemical-reduced films increased with the cooling temperature due to the reduced interference on spin magnetic moment caused by external thermal disturbances

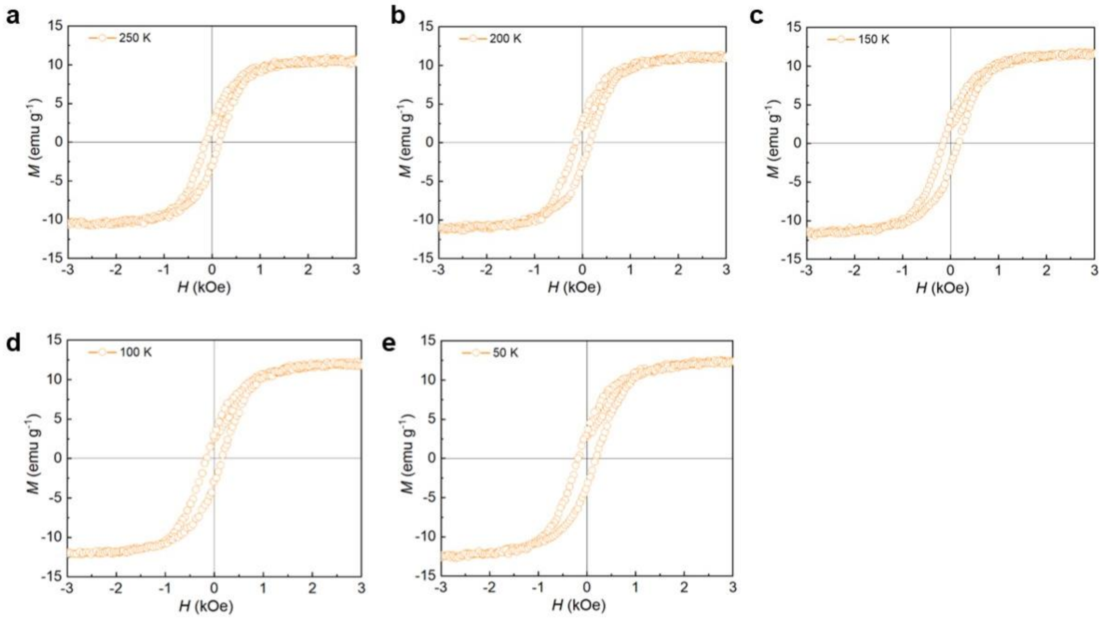

Supplementary Fig. 7 *M-H* curves at different temperature. **a** 250 K. **b** 200 K. **c** 150 K. **d** 100 K. **e** 50 K.

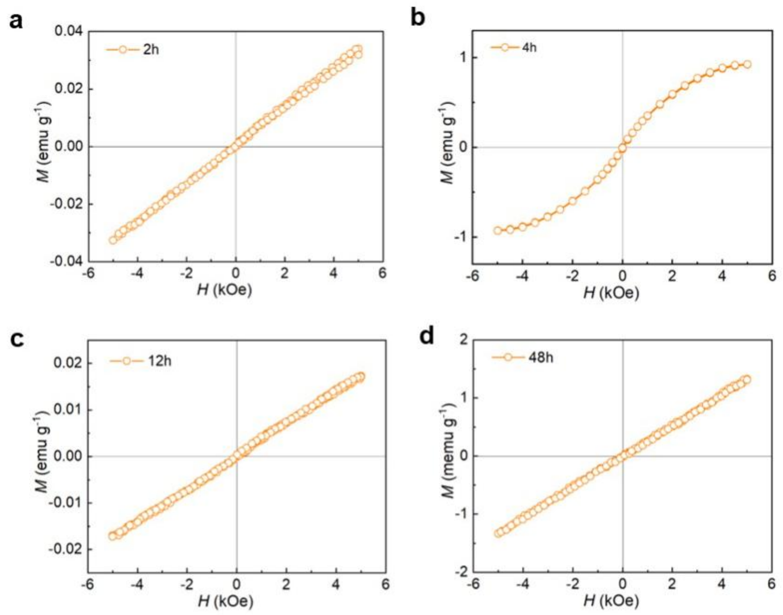

Supplementary Fig. 8 *M*-*H* curves of films reduced for 2h (a), 4h (b), 12h (c), 48h (d).

Due to its close-shell characterization, raw PDI films were diamagnetism. M_s of ferromagnetic films got weaker in glove box because of trace amounts of water and oxygen. PDI radical anions were oxidized into neutral PDI after exposing to air, and the films show diamagnetism. After high-temperature ablation, PDI species decomposed into carbon and exhibited diamagnetism, indicating that the ferromagnetism came out from PDI radicals.

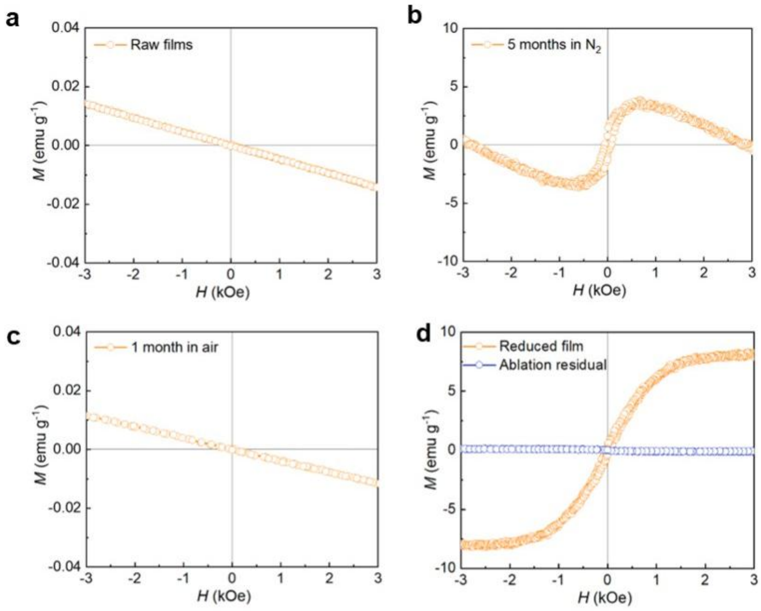

Supplementary Fig. 9 Magnetic properties after exposure in air and high-temperature digestion. **a** Raw films presented a diamagnetic *M-H* curve **b** Topochemical-reduced films show weaker ferromagnetism after 5 months in N_2 glove box. **c** Reduced films stored 30 days in air, exhibiting diamagnetism. **d** *M-H* curve of the reduced film and the ablation residual, the latter presenting diamagnetism.

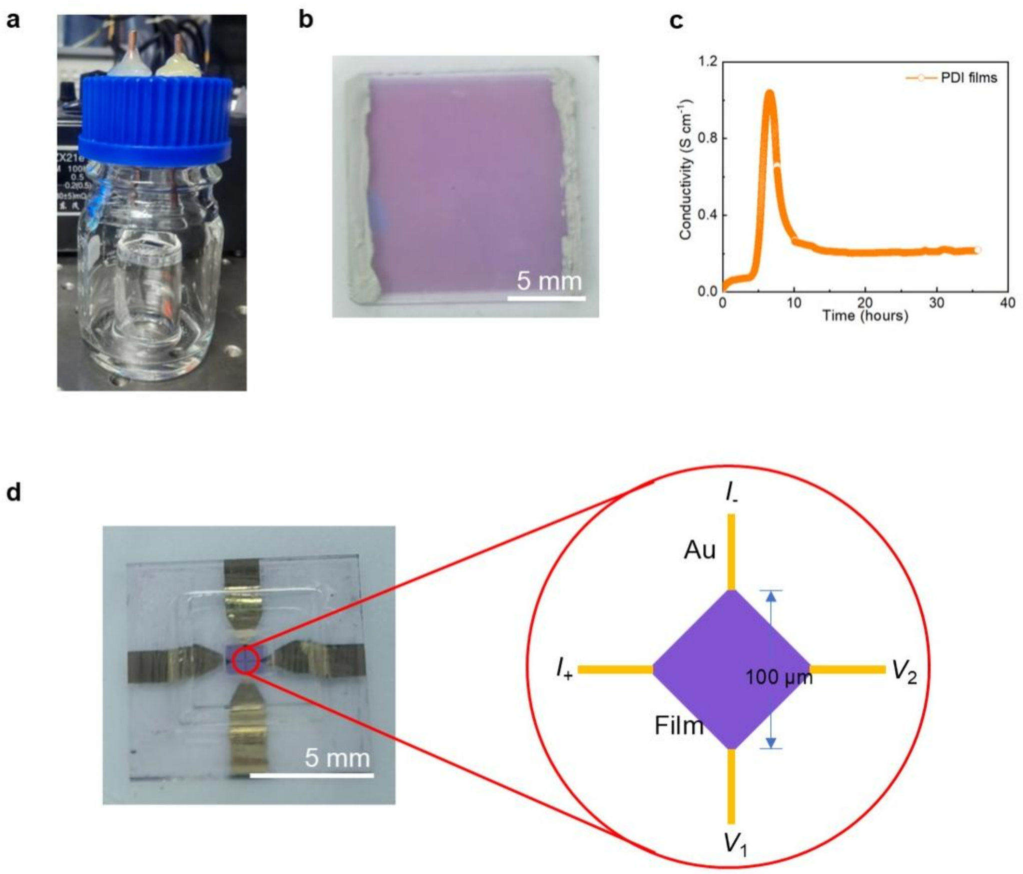

Supplementary Fig. 10 Electrical properties tests. **a** Homemade bottles used to monitor the conductivity while treating films. **b** Electrode configuration of *R*-*t* tests. **c** In site test of conductivity of the film during treatment measured by Keithley 2700 in a N_2 glove box. **d** Electrode configuration of four-point probe method in *R*-*T* tests. The distance between the opposite electrodes was100 µm.

100 nm NDI neutral films were prepared by thermal evaporation. The films had high crystallinity and orientation. NDI molecules had a face-on orientation on the substrate.

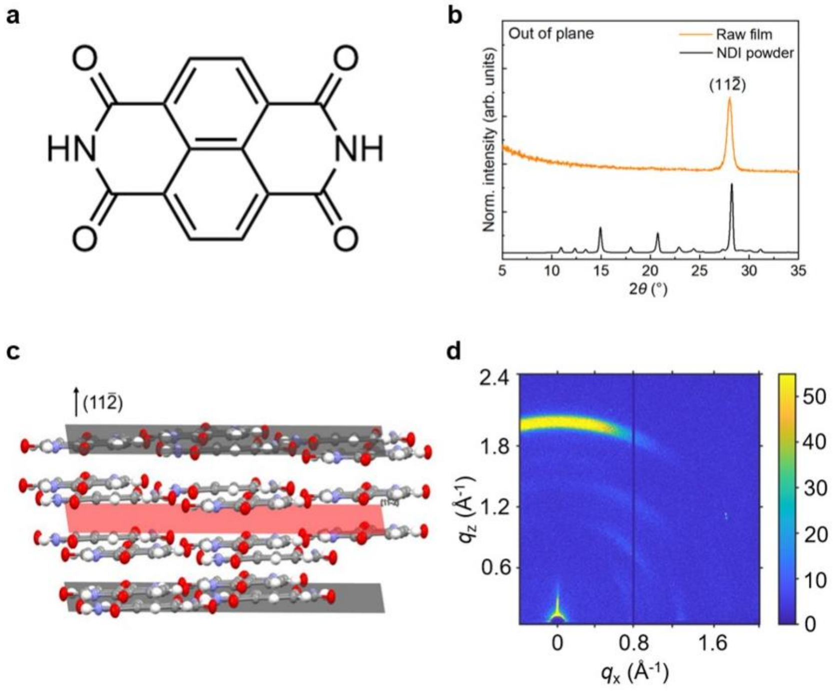

Supplementary Fig. 11 Structure of raw NDI films. **a** Molecular formula of NDI. **b** Comparison of GI-XRD patterns of raw films and Powder-XRD patterns of NDI. The peak of the two correspond well, indicating that the films are composed of NDI polycrystals. Molecular arrangements on crystal planes extracted from NDI single crystal and powder diffraction data are presented in **c** (11$\bar{2}$). **d** GIWAXS pattern of raw NDI films. Raw films exhibit face-on orientation.

We captured the EPR signal of the films reduced for different time. The intensity increased for the first 6 h and then decreased. So we chose 6 h to be the best reducing time. UV spectra confirmed the existence of ionized NDI in the films.

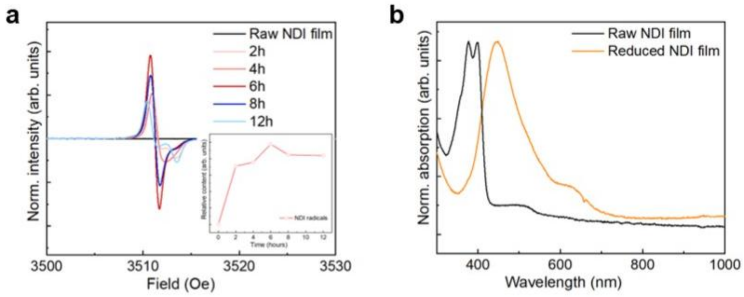

Supplementary Fig. 12 Composition of reduced NDI film. **a** EPR spectra of raw films and films reduced for 2 h, 4 h, 6 h, 8h, 12 h. Inset: Changes of relative content of radicals in the films over reducing time. **b** UV-vis spectra of raw films and films reduced for 6 h.

After topochemical reduction, the films show weaker crystallinity and orientation. The $(11\bar{2})$ peak shifted to smaller angle with a π-π distance of 3.26 Å, in agreement with the NDI radical powder reported before. The appearance of the signal at $q_z \approx 0.73$ Å^{-1} (10.3° in GI-XRD) can be assigned to the lamellar stacking of NDI molecules.

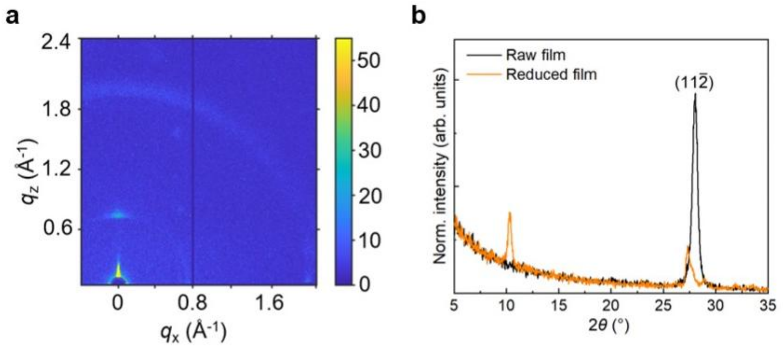

Supplementary Fig. 13 Changes in structure of NDI film after topochemical reduction. **a** GIWAXS pattern of reduced films with a weaker face-on orientation. **b** Comparison of XRD pattern between raw films and reduced films. The crystallinity decreased, and the peak at 28.05°in raw films moved to 27.30° in reduced films, implying the π-π distance changed 3.18 Å from to 3.26 Å.

Topochemical-reduced NDI films had a room-temperature ferromagnetism of 10.0 emu/g and a coercivity of 64.3 Oe, while that at 10 K was 11.8 emu/g and 78.6 Oe, respectively.

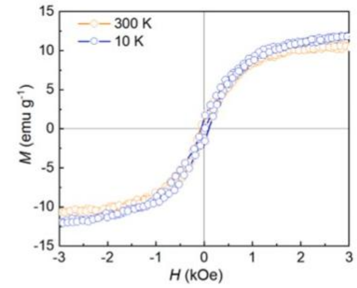

Supplementary Fig. 14 Magnetic properties of NDI radical films. *M-H* hysteresis loops of NDI films reduced in the same way taken at 300 K and 10 K, exhibiting an M_s of 10.0 emu/g at room-temperature and 11.8 emu/g at 10 K.

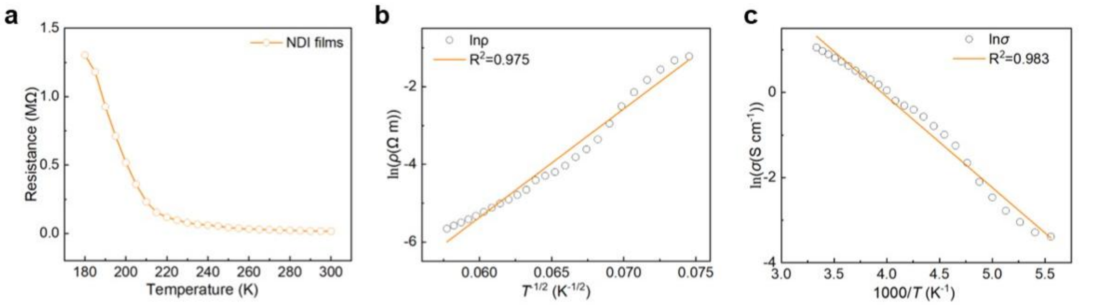

Supplementary Fig. 15 Semiconductivity of reduced NDI films. **a** Film resistance at different temperature. As the temperature decreases, the resistance increases exponentially, showing a typical characteristic of semiconductors. **b** The relationship between lnρ and $T^{-1/2}$ indicates a 1D-VRH conduction model in the film. **c** The relationship between natural logarithm of conductivity (lnσ) and $1/T$, and the result of linear fitting gives an apparent activating energy of 183.7 meV.

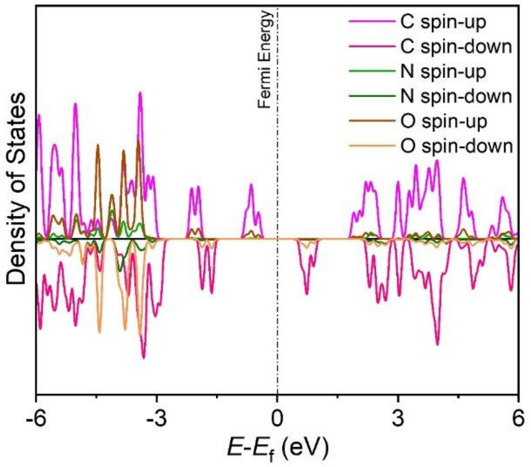

Supplementary Fig. 16 Atom-projected density of states (PDOS) analysis on the optimized H-doped PDI primitive cell.

The spin density distribution of radicals was given through M05-2X/6-31+G(d).

Based on the above research, we believe that the key to designing room temperature organic ferromagnetic semiconductors is to first select radical molecules with large π-conjugated structures to achieve moderate orbital overlap; Secondly, by precisely regulating the molecular packing, spin interactions that are conducive to ferromagnetic coupling can be achieved; Finally, by constructing a long-range ordered molecular assembly system, the ferromagnetic phase is thermodynamically stabilized. This strategy provides important guidance for the development of new room temperature organic magnetic materials.

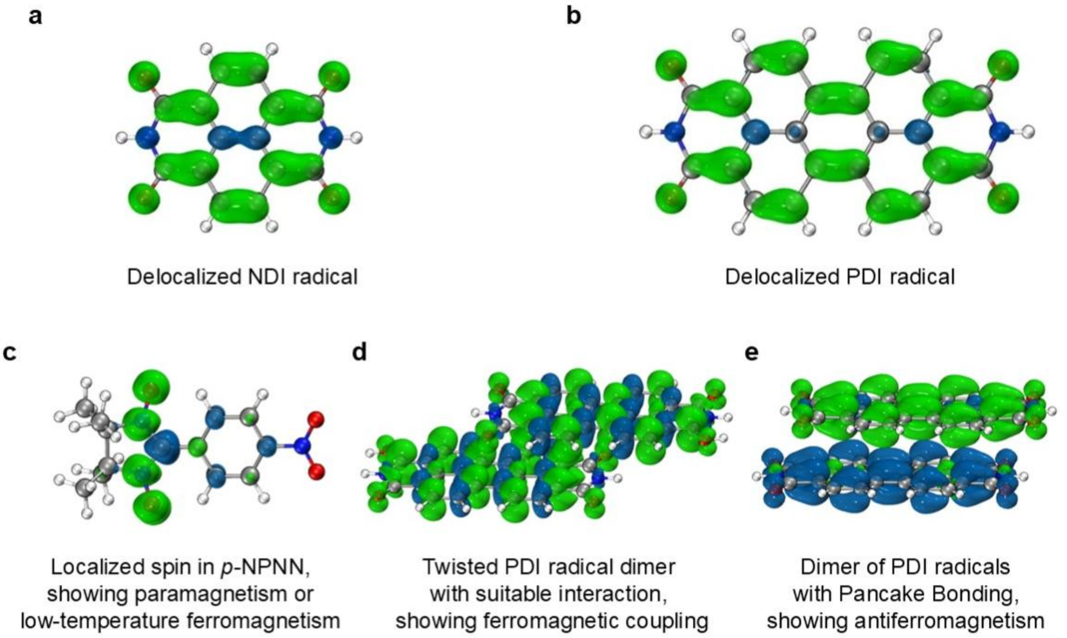

Supplementary Fig. 17 Guidance for the development of new room temperature organic magnetic materials. **a** Spin density distribution of NDI radical. **b** Spin density distribution of PDI radical. **c** Spin density distribution of *p*-NPNN. **d** Dimer of PDI radicals with 'Pancake Bonding' configuration. **e** Dimer of PDI radicals with ferromagnetic coupling.

Supplementary Table 1. Orientation degree and Herman's parameter of crystal plane $(12\bar{2})$ in raw films and topochemical-reduced films

	Raw film	Topochemical-reduced film
FWHM	58.0	89.9
Π (Orientation degree)	67.8%	50.0%
f (Herman's parameter)	-0.276	-0.162

Two approaches[6] were employed to describe the crystal orientation: the degree of orientation (Π) and Herman's orientation parameter (f)

$$\Pi = \frac{180 - FWHM}{180} * 100\%$$

$$f = \frac{3\langle cos^2\theta\rangle - 1}{2}$$

$$\langle cos^2\theta\rangle = \frac{\sum_0^{\pi/2} I(\theta) sin\theta\, cos^2\theta}{\sum_0^{\pi/2} I(\theta) sin\theta}$$

θ is the azimuthal angle and $I(\theta)$is the intensity along the Debye-Scherrer ring.

The degree of orientation (Π) formula estimates the orientation by the full width at half-maximum (FWHM) measurements of the peaks in a plot of intensity versus azimuthal angle at the relevant value of 2θ. The value of f ranges between -0.5 and 1 for perfect orientation perpendicular to and parallel to the fiber direction, respectively. Random orientation yields a value of f of 0.

After topochemical reduction the orientation of plane $(12\bar{2})$ in the films decreased.

Supplementary Table 2. Metal impurities analysis by ICP-MS for sublimated neutral PDI powders.

Element	Content (ppm)
Fe	11.5
Co	None
Ni	1.12
Mn	0.408

To provide a conservative upper-bound estimate, we assume the most extreme scenario in which all Fe impurities contribute fully to ferromagnetism. Using the bulk saturation magnetization of metallic Fe (approximately 218 emu g^{-1}, corresponding to ~2.2 μ_B per Fe atom) and Ni (approximately 55 emu g^{-1}, corresponding to ~0.6 μ_B per Ni atom), the maximum possible contribution from Fe impurities can be estimated as:

$$M_s^{impurity} \leq 218 \times 11.5\times10^{-6} + 55 \times 1.12\times10^{-6} = 2.6\times10^{-3} \text{ emu g}^{-1}$$

Supplementary Table 3. Metal impurities analysis by ICP-MS for topochemical-reduced PDI films.

Element	Content (ppm)
Fe	<15
Co	None
Ni	None
Mn	None

$$M_s^{impurity} \leq 218 \times 15\times10^{-6} = 3.3\times10^{-3} \text{ emu g}^{-1}$$

Metal impurities in reduced PDI films can only provide an M_s of 3.3×10^{-3} emu/g , ruling out the interference of impurities on the results.

Supplementary Table 4. The M_s and T_c in purely organic magnets.

Material	M_s (emu/g)	T_c (K)	Ref.
π-conjugated polymer	0.002	10	7
S-N radical	0.03	36	8
TDAE-C_{60}	0.84	17.5	9
p-NPNN	10.1	0.6	10
Pyrene-F4TCNQ	0.0016	305	11
p-TCNQ	0.0025	495	12
Coronene-TCNQ	0.0037	>300	13
Hydrogenated C_{60} film	0.17	>300	14
Porous Organic Radical Frameworks	0.4	465	15
NDI radical aggregates	1.1	>300	16
PDI radical aggregates	1.2	>400	17
PDI radical film	10.5	>300	This work

Supplementary Table 5. Metal impurities analysis by ICP-MS for reduced NDI films.

Element	Content (ppm)
Fe	<30
Co	None
Ni	None
Mn	None

$$M_s^{\text{impurity}} \leq 218 \times 30\times10^{-6} = 6.6\times10^{-3}\ \text{emu g}^{-1}$$

Metal impurities in reduced NDI films can only provide an M_s of 6.6×10^{-3} emu/g , ruling out the interference of impurities on the results.

Supplementary Table 6. Twist angle and slip distance for the pristine neutral PDI and the H-doped PDI structures.

Structure	dx (Å)	dy (Å)	θ (°)
pristine PDI	3.34	1.10	0.0
H-doped PDI	5.90	0.58	4.5

Supplementary references

1. Casasanta, G., Falcini, F. & Garra, R. Beer–Lambert law in photochemistry: A new approach. *Journal of Photochemistry and Photobiology A: Chemistry* **432**, 114086 (2022).
2. Amalina, A. N. *et al.* Shedding light on N719 dye adsorption mechanisms on Degussa (Evonik) P25 TiO_2 nanoparticles towards dye-sensitized solar cell performance. *Powder Technol.* **446**, 120087 (2024).
3. Al-Salihi, K. J. J. Evaluation of Dye Loading on Photoanodes of Dye-Sensitized Solar Cells Utilizing a Mixture of TiO_2 and Magnesium/Aluminum Layered Double Hydroxide (LDH). *J. Electron. Mater.* **53**, 6012-6022 (2024).
4. Chaudhari, A., Kumar, A., Kumar, S. & Kushwaha, S. Synthesis of TiO_2 nanoparticles by green approach: Application as photoanode for dye-sensitized solar cells. *Mater. Res. Bull.* **179**, 112909 (2024).
5. Dang, V. Q. & Teets, T. S. A practical guide to measuring and reporting photophysical data. *Dalton Trans.* **54**, 16268-16284 (2025).
6. Peng, J., Ellingham, T., Sabo, R., Turng, L.-S. & Clemons, C. M. Short cellulose nanofibrils as reinforcement in polyvinyl alcohol fiber. *Cellulose* **21**, 4287-4298 (2014).
7. Rajca, A., Wongsriratanakul, J. & Rajca, S. Magnetic Ordering in an Organic Polymer. *Science* **294**, 1503-1505 (2001).
8. Banister, A. J. *et al.* Spontaneous Magnetization in a Sulfur–Nitrogen Radical at 36 K. *Angew. Chem. Int. Ed. Engl.* **35**, 2533-2535 (1996).
9. Tanaka, K. *et al.* Magnetic properties of TDAE-C_{60} and TDAE-C_{70}, where TDAE is tetrakis(dimethylamino)ethylene. *Phys. Rev. B* **47**, 7554-7559 (1993).
10. Nakazawa, Y. *et al.* Low-temperature magnetic properties of the ferromagnetic organic radical, p-nitrophenyl nitronyl nitroxide. *Phys. Rev. B* **46**, 8906-8914 (1992).
11. Yang, Y., Wei, M. & Qin, W. Polarized spin-photon coupling in organic ferromagnetic magneto-optic crystals. *Appl. Mater. Today* **25**, 101229 (2021).
12. Mahmood, J. *et al.* Organic Ferromagnetism: Trapping Spins in the Glassy State of an Organic Network Structure. *Chem* **4**, 2357-2369 (2018).
13. Wei, M. *et al.* Organic Multiferroic Magnetoelastic Complexes. *Adv. Mater.* **32**, 2003293 (2020).
14. Makarova, T. L. *et al.* Laser controlled magnetism in hydrogenated fullerene films. *J. Appl. Phys.* **109**, 083941 (2011).
15. Phan, H. *et al.* Room-Temperature Magnets Based on 1,3,5-Triazine-Linked Porous Organic Radical Frameworks. *Chem* **5**, 1223-1234 (2019).
16. He, X. *et al.* Magnetic Properties of Self-Assemble Naphthalene Diimide Radical Aggregates. *Small* **20**, 2311766 (2024).
17. Jiang, Q. *et al.* Room-Temperature Ferromagnetism in Perylene Diimide Organic Semiconductor. *Adv. Mater.* **34**, 2108103 (2022).
