# Supplementary material for: Achieving high-performance room-temperature organic ferromagnetic semiconductor films via topochemical reduction
Source: Nat Commun. 2026 Apr 15;17:5197. doi: 10.1038/s41467-026-71866-2 (PMC13253818; doi:10.1038/s41467-026-71866-2)
Supplement: Supplementary file 2 — Transparent Peer Review file [file 41467_2026_71866_MOESM2_ESM.pdf]

nature portfolio

Peer Review File

Achieving High-Performance Room-Temperature Organic Ferromagnetic Semiconductor Films via Topochemical Reduction

Corresponding Author: Professor Yuguang Ma

This file contains all reviewer reports in order by version, followed by all author rebuttals in order by version.

Version 0:

Reviewer comments:

Reviewer #1

(Remarks to the Author)
The manuscript reports a meaningful advancement in the development of high-performance organic ferromagnetic semiconductors. Although this field has long been constrained by the inherent coupling between radical formation and structural ordering, the authors' topochemical reduction strategy provides an important conceptual and practical breakthrough. By decoupling these two processes, the work delivers PDI and NDI radical films that simultaneously exhibit strong room-temperature ferromagnetism and semiconducting behavior—an achievement of substantial significance. This study offers new ideas and methodological guidance for future organic spintronic materials. If the authors can clarify and strengthen the points below, the manuscript will be suitable for publication with minor revisions.
1. The manuscript states that metal impurities "can only provide 2.6×10^{-3}–3.3×10^{-3} emu g^{-1}" of magnetization. This is an important quantitative claim, but the current explanation is insufficient. Please provide a complete impurity-based Ms calculation.
2. The temperature-dependent conductivity is fitted using only the 1D-VRH model. Whether 2D-VRH, 3D-VRH, Arrhenius models were considered, and why 1D-VRH is physically justified for a polycrystalline thin film. A detailed discussion of trap-assisted hopping, disorder-induced localized states, or dopant-induced subgap states may be necessary to reconcile these values and clarify the conduction mechanism.
3. XMCD spectra must be obtained from the difference between XAS recorded under opposite circular polarizations. Please clarify: whether the measurement used true left- and right-circularly polarized X-rays, or whether it used field-reversal under a fixed polarization. If the latter, please justify equivalence and discuss any limitations. A short methodological clarification will strengthen confidence in the magnetic origin assignment.
4. To substantiate the claim of precise structural control via topochemical reduction, it is essential that the authors quantitatively tabulate and directly compare the twist angle and slip distance for both the pristine neutral PDI structure and the optimized H-doped PDI radical structure.
5. In addition to presenting the information on the band gap and exchange splitting, please perform a Partial Density of States (PDOS) analysis. This is crucial for determining whether the spin density predominantly originates from the p orbitals of the C atoms (perylene core) or the N atoms (imide moiety). Such detailed analysis will provide crucial theoretical evidence to validate and complement the experimental XMCD results.
After addressing the points above, the manuscript will be suitable for publication in Nature Communications.

Reviewer #2

(Remarks to the Author)
The authors report organic ferromagnetic semiconductor films based on perylene diimide (PDI) and naphthalene diimide (NDI) prepared via a topochemical reduction strategy. By reducing pre-ordered evaporated films with hydrazine vapor, they aim to decouple radical generation from structural organization, obtaining strongly coupled π-radical assemblies with shortened π–π stacking distances (~3.26 Å), room-temperature ferromagnetism (Ms ≈ 10.5 emu g^{-1}) and semiconducting transport. They further support their claims with GIWAXS/GI-XRD, EPR, UV-Vis, PPMS magnetometry, XMCD, conductivity measurements and DFT calculations. The manuscript presents promising results on room-temperature organic ferromagnetic semiconductor films and a potentially useful topochemical reduction strategy. However, the claims regarding

intrinsic ferromagnetism, magnitude of Ms, and general design principles are not fully supported by the data and analysis presented. I therefore recommend major revision, with substantial strengthening of the experimental evidence and clarification of the conceptual advances as outlined below.

1. The authors present this work as a “paradigm-shifting” topochemical reduction strategy and a “universal method” to obtain high-performance organic ferromagnetic semiconductors. However, the differences in scientific advance compared to previous PDI-based ferromagnets (Ref. 12) are not convincingly articulated.
2. At present, the narrative that topochemical reduction “decouples radical generation from structural assembly” feels more descriptive than mechanistically supported. The authors should clarify what cannot be achieved with previous strategies and why this process is fundamentally different, beyond a shorter π–π distance by a few hundredths of an Å. In this context, the statement that “the shorter π–π distance of 3.26 Å (vs. 3.30 Å reported previously) may be the critical factor enhancing spin–spin interactions. By decoupling radical generation from structural assembly, the topochemical reduction strategy enables long-range ordered packing of strongly conjugated radicals, which in turn promotes excellent magnetic properties” appears largely speculative and is not convincingly verified by the current data. If the authors wish to retain this argument, they should provide direct experimental or computational support that a 0.04 Å change in π–π distance leads to a quantitatively meaningful enhancement in exchange interactions, and this effect is uniquely enabled by the proposed decoupling of radical generation from structural assembly.
3. The UV–Vis spectra indicate the coexistence of neutral, radical anion, and dianion species, but this point remains largely qualitative. For the “optimal” 6 h condition, the authors should estimate, even approximately, the relative fractions of radical anions, dianions, and neutrals, and discuss how sensitive the observed Ms and conductivity are to this balance. Ideally, Ms, radical concentration, and conductivity should be correlated as a function of reduction time to establish a clearer structure–property relationship.
4. I have concerns about how the structural information from pristine PDI powder and single-crystal data is being used as a proxy for the reduced films. Because the key step here is a topochemical reduction, it is not obvious that the molecular packing and layer stacking in the post-reduction films remain identical to those of the unreduced PDI powder or single crystal. In particular, the change in the (020) reflection after reduction suggests that the corresponding d-spacing and/or stacking motif may have been altered, so it is not clear that one can safely state that the d-spacing is “the same” as in the pristine structure. More generally, I do not see solid evidence that the alignment and packing of the reduced films are structurally identical to those in the pre-reaction PDI powder and single-crystal data, and the authors do not provide a CIF file or other crystallographic details that would allow readers to critically evaluate the proposed structure.
5. Separately, the AFM data themselves raise additional concerns, as the authors describe a decrease in crystallinity and orientation after reduction. In light of this, it becomes even more important to provide direct structural evidence for the reduced material (e.g. powder XRD or single-crystal data on the reduced compound, or at least a more rigorous analysis of the film GIWAXS/GI-XRD patterns) rather than relying on the pristine PDI structure as a template. Related to this, the structural model in Fig. 1e involving “insertion of proton” into the lattice appears highly speculative in the absence of direct proof.
6. Supplementary Fig. 12 shows an apparently enhanced orientation of the diffraction feature around 10° after reduction, but this is neither described nor interpreted in the main text or the Supplementary Information, and an explicit interpretation of this feature is clearly needed.
7. More fundamentally, it is not obvious that the procedure used to determine the film mass is sufficiently robust for a quantitative Ms value. Dissolving a reduced film and inferring its mass from the absorption of the resulting solution implicitly assumes complete and reproducible dissolution, a well-defined chemical composition (neutral/radical/dianion, counterions) that matches the calibrated molar absorptivity, and no material loss during transfer or handling. In a system where the active phase is a mixed-valence radical salt obtained by topochemical reduction, these assumptions are far from trivial. Any small underestimation of the mass (e.g. due to incomplete dissolution or incorrect extinction coefficients for the reduced species) will directly lead to an overestimation of Ms in emu g^{-1}.

Version 1:

Reviewer comments:

Reviewer #1

(Remarks to the Author)
The authors have responded to all questions raised by the reviewers, which can be published at this stage.

Reviewer #2

(Remarks to the Author)
The authors report high-performance organic ferromagnetic semiconductor films based on perylene diimide (PDI) and naphthalene diimide (NDI) prepared via a topochemical reduction strategy. By reducing pre-ordered evaporated films with hydrazine vapor, they decouple radical generation from structural organization and obtain strongly coupled π-radical assemblies with shortened π–π stacking distances (~3.26 Å), robust room-temperature ferromagnetism (Ms ≈ 10.5 emu g^{-1}), and semiconducting transport. In the revised manuscript and rebuttal, the authors substantially strengthened the experimental support for these claims, including additional time-dependent EPR/magnetometry/conductivity correlations, clearer and more appropriately qualified structural interpretation from GIWAXS/GI-XRD, and improved justification for the intrinsic nature of the ferromagnetism (including impurity considerations). The cross-validation using multiple complementary probes (PPMS magnetometry, XMCD, spectroscopy, and DFT) now provides a coherent and convincing picture of

topochemically generated, strongly interacting organic radical solids that exhibit room-temperature ferromagnetism with meaningful electronic transport.

Overall, I find that my major concerns regarding the magnitude and origin of Ms, the relationship between reduction time/radical concentration/transport, and the conceptual framing of the strategy have been addressed satisfactorily. I therefore consider the manuscript acceptable for publication.

Open Access This Peer Review File is licensed under a Creative Commons Attribution 4.0 International License, which permits use, sharing, adaptation, distribution and reproduction in any medium or format, as long as you give appropriate credit to the original author(s) and the source, provide a link to the Creative Commons license, and indicate if changes were made.

In cases where reviewers are anonymous, credit should be given to 'Anonymous Referee' and the source.

The images or other third party material in this Peer Review File are included in the article's Creative Commons license, unless indicated otherwise in a credit line to the material. If material is not included in the article's Creative Commons license and your intended use is not permitted by statutory regulation or exceeds the permitted use, you will need to obtain permission directly from the copyright holder.

To view a copy of this license, visit https://creativecommons.org/licenses/by/4.0/

Responses to the reviewers' comments

We sincerely appreciate the editor and reviewers for their valuable feedback. We have examined all the comments carefully and addressed your concerns in the revised manuscript. You will find our point-by-point responses to all the comments in blue below and the additions have been marked in yellow in the revised manuscript.

****

Reviewer 1:

The manuscript reports a meaningful advancement in the development of high performance organic ferromagnetic semiconductors. Although this field has long been constrained by the inherent coupling between radical formation and structural ordering, the authors' topochemical reduction strategy provides an important conceptual and practical breakthrough. By decoupling these two processes, the work delivers PDI and NDI radical films that simultaneously exhibit strong room temperature ferromagnetism and semiconducting behavior—an achievement of substantial significance. This study offers new ideas and methodological guidance for future organic spintronic materials. If the authors can clarify and strengthen the points below, the manuscript will be suitable for publication with minor revisions.

Response: We sincerely thank your positive evaluation. In response to your comments, we have revised the Manuscript and Supplementary Information accordingly, including additional calculations, expanded discussions, and clearer methodological descriptions.

1. The manuscript states that metal impurities "can only provide 2.6×10^{-3}–3.3×10^{-3} emu

g^{-1}" of magnetization. This is an important quantitative claim, but the current explanation is insufficient. Please provide a complete impurity based Ms calculation.

Response: We thank you for pointing out the need for a clearer and more quantitative explanation. We have now explicitly included the impurity-based saturation magnetization (M_s) estimation in the revised Supplementary Information.

ICP-MS analysis shows that the Fe content in the ferromagnetic PDI films is below 15 ppm, while Co, Ni, and Mn are absent within the detection limit (Supplementary Table 3). To provide a conservative upper-bound estimate, we assume the most extreme scenario in which all Fe impurities contribute fully to ferromagnetism. Using the bulk saturation magnetization of metallic Fe (approximately 218 emu g^{-1}, corresponding to ~2.2 μ_B per Fe atom), the maximum possible contribution from Fe impurities can be estimated as:

$$M_s^{impurity} \leq 218 \text{ emu g}^{-1} \times 15\times10^{-6} \approx 3.3\times10^{-3} \text{ emu g}^{-1}$$

This value represents an absolute upper limit and is more than four orders of magnitude smaller than the experimentally measured saturation magnetization of the PDI radical films (10.5 emu g^{-1}). Therefore, metal impurities cannot account for the observed ferromagnetism.

A similar analysis was performed for the reduced NDI films, where the Fe content is below 30 ppm (Supplementary Table 5), yielding an upper-limit impurity contribution of approximately 6.6×10^{-3} emu g^{-1}. This value is likewise negligible compared with the measured M_s of the NDI radical films.

These quantitative estimates conclusively rule out metal impurities as the origin

of the observed ferromagnetism in both PDI and NDI films. Corresponding clarifications have been added to the revised Supplementary Information.

Supplementary Table 2. Metal impurities analysis by ICP-MS for sublimated neutral PDI powders.

Element	Content (ppm)
Fe	11.5
Co	None
Ni	1.12
Mn	0.408

To provide a conservative upper-bound estimate, we assume the most extreme scenario in which all Fe impurities contribute fully to ferromagnetism. Using the bulk saturation magnetization of metallic Fe (approximately 218 emu g^{-1}, corresponding to ~2.2 μ_B per Fe atom) and Ni (approximately 55 emu g^{-1}, corresponding to ~0.6 μ_B per Ni atom), the maximum possible contribution from Fe impurities can be estimated as:

$$M_s^{impurity} \leq 218 \times 11.5\times10^{-6} + 55 \times 1.12\times10^{-6} = 2.6\times10^{-3} \text{ emu g}^{-1}$$

Supplementary Table 3. Metal impurities analysis by ICP-MS for topochemical-reduced PDI films.

Element	Content (ppm)
Fe	<15
Co	None
Ni	None
Mn	None

$$M_s^{impurity} \leq 218 \times 15\times10^{-6} = 3.3\times10^{-3} \text{ emu g}^{-1}$$

Metal impurities in reduced PDI films can only provide an M_s of 3.3×10^{-3} emu/g , ruling out the interference of impurities on the results.

Supplementary Table 5. Metal impurities analysis by ICP-MS for reduced NDI films.

Element	Content (ppm)
Fe	<30
Co	None
Ni	None
Mn	None

$$M_s^{impurity} \leq 218 \times 30\times10^{-6} = 6.6\times10^{-3} \text{ emu g}^{-1}$$

Metal impurities in reduced NDI films can only provide an M_s of 6.6×10^{-3} emu/g , ruling out the interference of impurities on the results.

2. The temperature dependent conductivity is fitted using only the 1D VRH model. Whether 2D VRH, 3D VRH, Arrhenius models were considered, and why 1D VRH is physically justified for a polycrystalline thin film. A detailed discussion of trap assisted hopping, disorder induced localized states, or dopant induced subgap states may be necessary to reconcile these values and clarify the conduction mechanism.

Response: We thank you for this helpful suggestion. In the revised Manuscript/Supplementary Information, we now clarify that several transport models

were examined for the temperature-dependent conductivity.

Specifically, the experimental data were fitted using the 1D-VRH, 2D-VRH, 3D-VRH, and Arrhenius models. The corresponding coefficients of determination (R^2) are 0.997, 0.989, 0.982, and 0.986, respectively (Response fig. 1). Among these models, the 1D-VRH model provides the best statistical agreement with the experimental data, and we therefore adopt it as the most appropriate phenomenological description of the measured transport behavior.

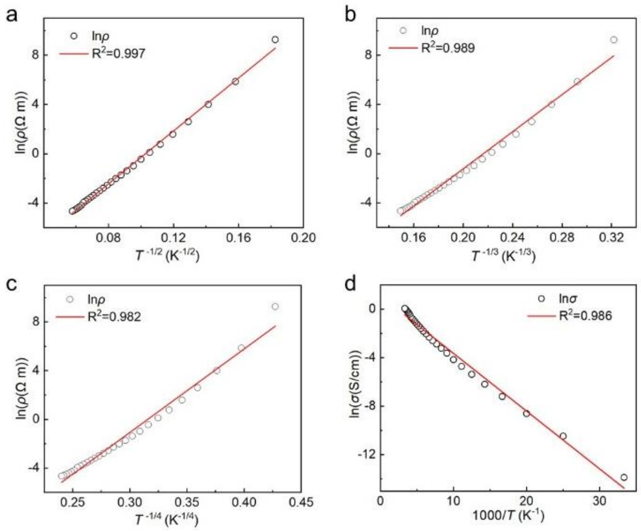

Response Fig.1 Linear fitting of 1D-VRH (a), 2D-VRH (b), 3D-VRH (c), and Arrhenius (d) models.

We emphasize that the resistance was measured in an in-plane electrode geometry. In polycrystalline and partially disordered films, charge transport can be dominated by hopping between localized states, with an effective dimensionality determined by the connectivity of the conducting network rather than the geometric dimensionality of the sample. In particular, spatial/energetic disorder, grain boundaries, trap-assisted hopping, and dopant-induced localized (subgap) states can constrain the dominant conduction pathways into quasi-chain-like percolative channels within the film plane, leading to an apparent 1D-VRH temperature dependence. We have added this discussion to better

justify the physical meaning of the 1D-VRH fitting in the manuscript.

In addition, although the Arrhenius model yields a slightly lower fitting quality, it still provides a useful apparent activation energy, which we report as a complementary descriptor of the semiconducting nature of the films. Corresponding text and fittings have been clarified in the revised version.

Revised text: "The data were fitted using the 1D-VRH, 2D-VRH, 3D-VRH, and Arrhenius models. The linear dependence of $T^{-1/2}$ and natural logarithm of resistivity (ln ρ) fits the best, confirming the 1D-VRH conduction model in the film (Fig. 3b).[23,24] The relationship between reciprocal temperature (1/T) and natural logarithm of electrical conductivity (ln σ), as described by the Arrhenius equation, gives a typical thermal excitation model for semiconductors with an apparent activation energy value at approximately 41 meV (Fig. 3c).[25]"

3. XMCD spectra must be obtained from the difference between XAS recorded under opposite circular polarizations. Please clarify: whether the measurement used true left and right circularly polarized X rays, or whether it used field reversal under a fixed polarization. If the latter, please justify equivalence and discuss any limitations. A short methodological clarification will strengthen confidence in the magnetic origin assignment.

Response: We thank you for this important methodological clarification. In our XMCD measurements, circularly polarized X-rays with a fixed helicity were used, and the XMCD signal was obtained by reversing the direction of the applied magnetic field (±

3000 Oe) during the XAS measurements.

This field-reversal approach is a well-established and widely accepted method for XMCD experiments, and it is formally equivalent to helicity reversal under the assumption of a linear and reversible magnetic response of the sample. The XMCD signal is defined as the difference between XAS spectra recorded with opposite relative orientations between the photon helicity and the sample magnetization, which can be achieved either by reversing the photon helicity or by reversing the magnetic field direction. Both approaches are commonly used in XMCD studies (*Nat. Phys.* **2024**, *20*, 281; *Interdiscip. Mater.* **2025**, *4*, 576; *Nat. Commun.* **2023**, *14*, 7063).

We have now clarified this experimental procedure and added the corresponding explanation in the methods section to avoid ambiguity.

Revised text: "C K-edge XAS and XMCD spectra of films were collected at beamline BL12B-a of the National Synchrotron Radiation Laboratory (NSRL), Hefei, and the signal of the total electron yield was measured at 300 K under a vacuum better than 10^{-7} Pa. XAS were recorded for 3 times under each magnetic-field direction (±3000 Oe) to ensure reproducibility, and the XMCD spectrum was obtained from the difference between the averaged XAS spectra measured under opposite fields. The XAS signals were processed using simple normalization as I/I_0 where I is the total electron yield signal of films and I_0 is the photocurrent from a gold mesh as a reference."

4. To substantiate the claim of precise structural control via topochemical reduction, it is essential that the authors quantitatively tabulate and directly compare the twist angle

and slip distance for both the pristine neutral PDI structure and the optimized H-doped PDI radical structure.

Response: We thank you for this suggestion. In response, we have quantified and directly compared the twist angle and slip distance for the pristine neutral PDI and the H-doped PDI structures, and the results are summarized in Supplementary Table 6.

Structure	dx (Å)	dy (Å)	θ (°)
pristine PDI	3.34	1.10	0.0
H-doped PDI	5.90	0.58	4.5

5. In addition to presenting the information on the band gap and exchange splitting, please perform a Partial Density of States (PDOS) analysis. This is crucial for determining whether the spin density predominantly originates from the p orbitals of the C atoms (perylene core) or the N atoms (imide moiety). Such detailed analysis will provide crucial theoretical evidence to validate and complement the experimental XMCD results.

Response: We thank you for this insightful suggestion. In response, we have performed an atom-projected density of states (PDOS) analysis to clarify the atomic origin of the spin polarization near the Fermi level. As shown by the spin-resolved atom-projected DOS, the states in the vicinity of the Fermi level are dominated by contributions from C atoms in the perylene π-core. The O atoms in the imide moiety exhibit only minor weight near the Fermi level, while the contribution from N atoms is essentially negligible. These results indicate that the spin polarization predominantly resides on the carbon-based π-framework.

This atom-projected PDOS analysis provides theoretical support for the

experimental XMCD measurements, which probe element-specific spin polarization. In particular, the dominant C contribution and the negligible N contribution near the Fermi level are consistent with our XMCD-based assignment of the spin-polarized electronic structure to the perylene π-core.

The corresponding PDOS results and discussion have been added and are shown in Supplementary Fig. 15.

Revised text: "The spin-resolved atom-projected DOS (PDOS) in Supplementary Fig. 16 shows that the states in the vicinity of the Fermi level are dominated by contributions from C atoms in the perylene π-core. The O atoms in the imide moiety exhibit only minor weight near the Fermi level, while the contribution from N atoms is essentially negligible. These results indicate that the spin polarization predominantly resides on the carbon-based π-framework, supporting for the experimental XMCD measurements."

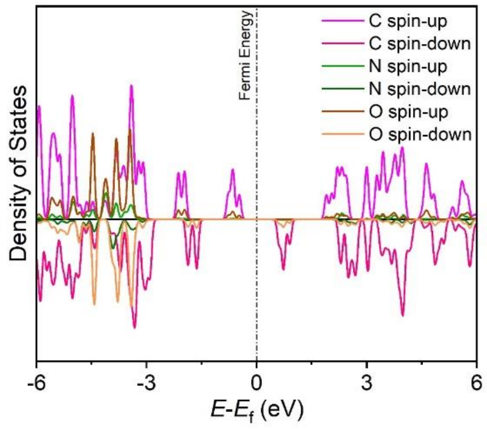

Supplementary Fig. 16 Atom-projected density of states (PDOS) analysis on the optimized H-doped PDI primitive cell.

****

Reviewer 2:

The authors report organic ferromagnetic semiconductor films based on perylene diimide (PDI) and naphthalene diimide (NDI) prepared via a topochemical reduction strategy. By reducing pre-ordered evaporated films with hydrazine vapor, they aim to decouple radical generation from structural organization, obtaining strongly coupled π-radical assemblies with shortened π-π stacking distances (~3.26 Å), room-temperature ferromagnetism (Ms ≈ 10.5 emu g^{-1}) and semiconducting transport. They further support their claims with GIWAXS/GI-XRD, EPR, UV-Vis, PPMS magnetometry, XMCD, conductivity measurements and DFT calculations. The manuscript presents promising results on room-temperature organic ferromagnetic semiconductor films and a potentially useful topochemical reduction strategy. However, the claims regarding intrinsic ferromagnetism, magnitude of Ms, and general design principles are not fully supported by the data and analysis presented. I therefore recommend major revision, with substantial strengthening of the experimental evidence and clarification of the conceptual advances as outlined below.

Response: We sincerely thank you for the careful and detailed assessment of our manuscript. In response to these comments, we have substantially revised the Manuscript and Supplementary Information. These revisions include additional quantitative analyses, expanded structure-property correlations, clearer distinctions between experimentally established results and mechanistic interpretations, and a more balanced discussion on the topochemical reduction strategy.

1. The authors present this work as a "paradigm-shifting" topochemical reduction strategy and a "universal method" to obtain high-performance organic ferromagnetic semiconductors. However, the differences in scientific advance compared to previous PDI-based ferromagnets (Ref. 12) are not convincingly articulated.

Response: We thank you for this important comment. Compared with previous work (Ref. 12), the significance of this study is fundamentally different.

In our previous work, we primarily demonstrated that ferromagnetism can be observed in organic radical semiconductors based on PDI systems. However, the measured saturation magnetization was 1.2 emu/g, only 9% of the theoretical maximum, indicating that only a small fraction of radicals participated in long-range ferromagnetic coupling. This result further corroborates our discussion in the Introduction that a major challenge remains in the field of organic ferromagnetic semiconductors: how to achieve long-range ordered packing in organic semiconductors while preserving ferromagnetic coupling between radicals. In practice, a trade-off must be made between intermolecular interactions and long-range ordered packing (we have modified Supplementary Fig. 1 for better illustration). For radicals with small conjugation, intermolecular interactions are weak, but long-range ordered molecular assembly is relatively easy to achieve, leading to high saturation magnetization only at low temperatures. In contrast, for radicals with large conjugation, intermolecular interactions are strong and Curie temperatures can exceed room temperature, but long-range ordered assembly is difficult to maintain, resulting in very low saturation magnetization, often only a small fraction of the theoretical maximum.

To address this challenge, in the present work, we propose the central strategy: Topochemical Reduction, to pre-construct a long-range ordered film of a large-conjugation neutral molecular semiconductor, and subsequently introduce radicals via topochemical reduction while preserving the original long-range structural order.

By decoupling structural organization from radical generation, this strategy enables a substantially larger fraction of radicals to participate in ferromagnetic coupling. As a result, the room-temperature saturation magnetization reaches 10.5 emu g^{-1}, corresponding to approximately 73% of the theoretical maximum, representing nearly an order-of-magnitude improvement over previously reported PDI-based system. By applying the same strategy, we achieved a saturation magnetization of 10.0 emu g^{-1} in NDI films, also representing nearly an order-of-magnitude improvement over previously reported NDI-based system, demonstrating the generality of this strategy.

This work therefore shifts the focus from demonstrating organic ferromagnetism to addressing how a large fraction of radicals can be coherently coupled in extended conjugated systems. We have revised the manuscript as proposed to better emphasize this conceptual advance and its distinction from our earlier work.

Revised text: "To address the challenge, we introduce topochemical reduction—a strategy involving in-situ chemical conversion on pre-ordered molecular films—that can decouple the generation of radicals from the construction of aggregate structures as a universal approach for fabricating high-performance organic ferromagnetic semiconductor films."

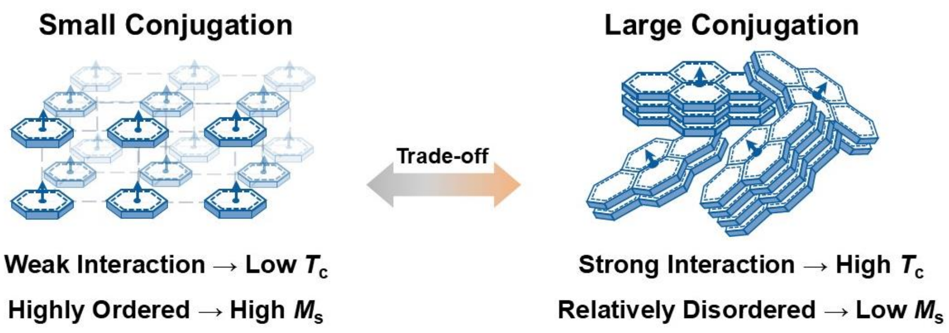

Supplementary Fig. 1 Conceptual trade-off between conjugation size, spin interaction, and achievable ferromagnetic performance in organic radical systems.

2. At present, the narrative that topochemical reduction "decouples radical generation from structural assembly" feels more descriptive than mechanistically supported. The authors should clarify what cannot be achieved with previous strategies and why this process is fundamentally different, beyond a shorter π–π distance by a few hundredths of an Å. In this context, the statement that "the shorter π–π distance of 3.26 Å (vs. 3.30 Å reported previously) may be the critical factor enhancing spin–spin interactions. By decoupling radical generation from structural assembly, the topochemical reduction strategy enables long-range ordered packing of strongly conjugated radicals, which in turn promotes excellent magnetic properties" appears largely speculative and is not convincingly verified by the current data. If the authors wish to retain this argument, they should provide direct experimental or computational support that a 0.04 Å change in π–π distance leads to a quantitatively meaningful enhancement in exchange interactions, and this effect is uniquely enabled by the proposed decoupling of radical generation from structural assembly.

Response: We thank you for this helpful critique and agree that our previous

wording was overly idealized and not mechanistically supported by the data. In particular, the statement that a ~0.04 Å shorter π-π distance is "the critical factor" and that topochemical reduction "decouples radical generation from structural assembly" in a uniquely verified way was too speculative.

As requested, we nonetheless performed an explicit periodic test by increasing the π-π separation by 0.04 Å. The computed electronic energies are E_{AFM} = −2435.34737765 Ry, and E_{FM} = −2435.35250162 Ry, giving $E_{AFM} - E_{FM}$ = +0.005124 Ry ≈ +70 meV (vs. +61 meV in the original structure). While this indicates a measurable distance sensitivity of the magnetic energetics, we acknowledge that it does not, by itself, substantiate the strong causal/mechanistic narrative in our original text; accordingly, our discussion has been revised to avoid an absolute interpretation.

We have therefore removed/rewritten this claim in the revised manuscript.

Revised text: "It is worth noting that compared with previous reports in which radical generation is accompanied by uncontrollable aggregation during solution-phase evaporation, topochemical reduction can decouple radical generation from structural assembly, pre-build the PDI molecules in the film in a well-organized state and obtain a high concentration of radicals in an orderly stacked ionic PDI film. Along with the shorter π-π distance of 3.26 Å, the films are expected to exhibit excellent magnetic properties."

3. The UV–Vis spectra indicate the coexistence of neutral, radical anion, and dianion species, but this point remains largely qualitative. For the "optimal" 6 h condition, the

authors should estimate, even approximately, the relative fractions of radical anions, dianions, and neutrals, and discuss how sensitive the observed Ms and conductivity are to this balance. Ideally, Ms, radical concentration, and conductivity should be correlated as a function of reduction time to establish a clearer structure–property relationship.

Response: We thank you for this important suggestion. We agree that correlating magnetic/electronic properties with the evolution of redox states during reduction would strengthen the structure-property relationship. In the revised Manuscript and Supplementary Information, we have clarified why UV-vis and XPS cannot provide a reliable quantitative three-component fraction in our system, and provided a time-dependent correlation among magnetization, relative radical concentration, and conductivity.

We attempted to quantify the relative fractions of the three redox states (neutral, radical anion, and dianion) by deconvoluting UV-vis absorption spectra acquired at different reduction times (Supplementary Fig. 4d). The reduction induces pronounced spectral evolution (band broadening and the emergence of a long-wavelength absorption tail extending into the near-infrared region), qualitatively supporting the formation of reduced species. However, the absorption bands at different reduction times remain heavily overlapped and do not show well-resolved, “fingerprint” peaks that can be uniquely assigned to the three states. The spectral changes are dominated by continuous broadening and tailing rather than separable bands; therefore, multi-component peak fitting / linear-combination deconvolution does not yield a unique, robust solution and is highly sensitive to baseline and fitting constraints. Consequently,

UV-vis alone cannot reliably deliver accurate quantitative ratios of the three species during the reduction process.

We also attempted to quantify the three species fractions by XPS (Supplementary Fig. 4e, f). Although high-resolution N 1s and O 1s spectra show clear evolution upon reduction (broadening and development of a tail/shoulder), the main peak positions remain essentially unchanged for reduced films (2-48 h), and no resolvable chemical-shift components can be reliably assigned to the three redox states. This is consistent with delocalized charge states in the conjugated framework, for which core-level binding-energy shifts are expected to be small and can be masked by line-shape changes rather than distinct peak separation. Constrained multi-peak fitting did not produce physically meaningful or stable fractions due to strong parameter correlation. Therefore, XPS likewise cannot provide accurate quantitative fractions of neutral/radical anion/dianion in our system.

While direct quantitative fractions of the three species cannot be robustly extracted from UV-vis/XPS, we agree that the reviewer's request for property correlation vs reduction time is essential. We therefore provide a consolidated time-dependent comparison using: EPR-derived relative radical concentration, *M-H* curves, and conductivity evolution.

We tracked the EPR signal as a function of reduction time and obtained the relative radical concentration by double integration in the Manuscript. The radical concentration increases during the early stage of reduction, reaches a maximum at ~6 h, and decreases upon further reduction due to conversion of EPR-active radical anions into EPR-silent

dianions (Supplementary Fig. 4a-c).

We have added *M-H* curves measured for films reduced for 2 h, 4 h, 12 h, and 48 h. These data show that the films reduced for 2 h, 12 h, and 48 h exhibit paramagnetic behavior in the measured field range, whereas the 4 h film shows a weak approach to saturation above ~2000 Oe with an almost negligible coercivity, consistent with the onset of extremely weak ferromagnetic coupling when a substantial radical population begins to form but before the coupling becomes robust. With further reduction to 6 h, the film exhibits a clear ferromagnetic hysteresis loop and a much more pronounced ferromagnetic response, indicating that strong ferromagnetism emerges only when the radical population and the coupling efficiency become sufficiently high.

The conductivity evolution during reduction was monitored in situ (Supplementary Fig. 10c) and the film conductivity at 6 h reaches ~0.90 S cm^{-1}. We speculate that the change in conductivity is due to changes in the structure of the doped and aggregated states of the film.

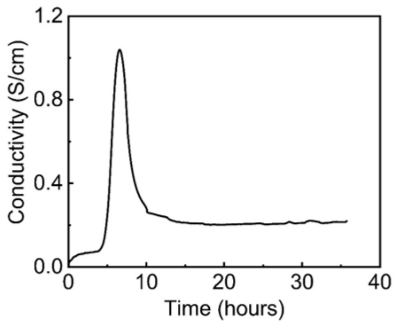

Supplementary Fig. 10 c In site test of conductivity of the film during treatment measured by Keithley 2700 in a N2 glove box.

Taken together, the added time-dependent magnetization data combined with the existing EPR-derived radical evolution and conductivity monitoring establish a

consistent correlation: early reduction increases radical population and yields predominantly paramagnetic behavior; intermediate reduction (e.g., 4 h) shows the onset of very weak ferromagnetic coupling; and the optimal 6 h condition (maximum radical population under our processing) produces robust room-temperature ferromagnetism together with semiconducting transport. We have revised the Supplementary Information to present this correlation and have added text in the revised Manuscript to clarify the sensitivity of magnetic/electronic properties to the reduction duration.

Finally, we note that a truly quantitative three-species fraction determination would require reference spectra (UV-vis/XPS) for pure neutral/radical anion/dianion states in the same film environment (including counterions and packing), or an orthogonal quantitative method (e.g., calibrated EPR spin counting with absolute standard combined with an independent determination of total reduced charge). This is an important direction for future work, but is beyond what can be robustly extracted from UV-vis/XPS alone in the present system.

Revised text: "The films treated with saturated steam of hydrazine hydrate solution for 6 hours show an absorption spectrum between the two above, with weak absorption appeared at 730, 816, and 976 nm, which were within the wavelength range of absorption of PDI radical anions reported in the literature.[17,18] We attempted to perform a quantitative analysis (Supplementary fig. 4e,f), but the results were not reliable. Ultimately, we use EPR data to validated that 6 hours represented optimal timing for obtaining films with maximum radical content under this process."

"We also measured the films reduced for different times (Supplementary Fig. 8). Those for 2 h, 12 h, and 48 h exhibit paramagnetic behavior in the measured field range, whereas the 4 h film shows a weak approach to saturation above ~2000 Oe with an almost negligible coercivity, consistent with the onset of extremely weak ferromagnetic coupling when a substantial radical population begins to form but before the coupling becomes robust indicating that strong ferromagnetism emerges only when the radical population and the coupling efficiency become sufficiently high."

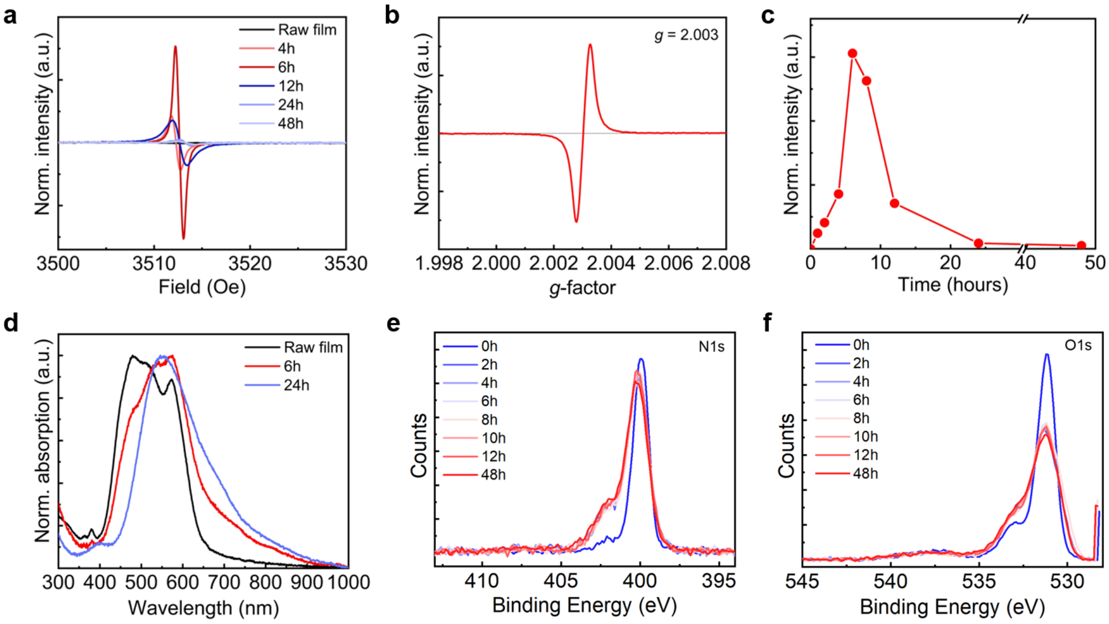

Supplementary Fig. 4 Film composition. **a** EPR spectra of raw films and films reduced for 4h, 6h, 12h, 24h, 48h. **b** *g*-factor of the electrons in the films. **c** Changes of relative concentration of radicals in the films over reducing time. **d** UV-vis spectra of raw films and films reduced for 6h, 24h. Binding energy of N1s (**e**) and O1s (**f**) electrons in films with different reduction time.

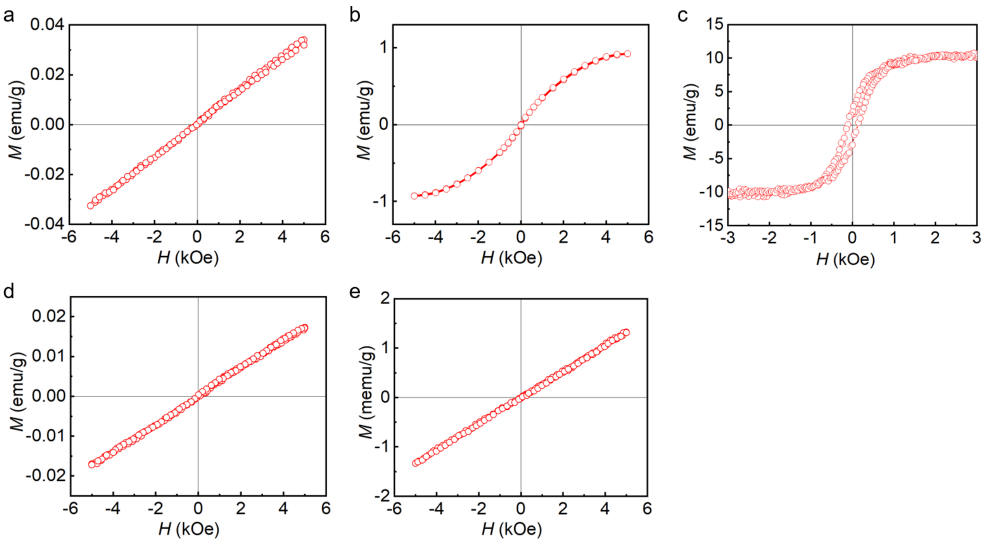

Supplementary Fig. 8 *M-H* curves of films reduced of 2h (a), 4h (b), 6h (c), 12h (d), 48h (e).

4. I have concerns about how the structural information from pristine PDI powder and single-crystal data is being used as a proxy for the reduced films. Because the key step here is a topochemical reduction, it is not obvious that the molecular packing and layer stacking in the post-reduction films remain identical to those of the unreduced PDI powder or single crystal. In particular, the change in the (020) reflection after reduction suggests that the corresponding d-spacing and/or stacking motif may have been altered, so it is not clear that one can safely state that the d-spacing is "the same" as in the pristine structure. More generally, I do not see solid evidence that the alignment and packing of the reduced films are structurally identical to those in the pre-reaction PDI powder and single-crystal data, and the authors do not provide a CIF file or other crystallographic details that would allow readers to critically evaluate the proposed structure.

Response: We thank you for this important comment. We agree that our original

wording may have given the impression that the structure of the reduced films is identical to that of pristine PDI, and we have revised the manuscript to clarify this point.

First, we emphasize that we do not claim the reduced films to have the same crystal structure as pristine PDI. Instead, the pristine PDI structure is used as a reference framework to assist the interpretation of diffraction features.

Second, the topochemically reduced films are polycrystalline films with mixed molecular valence. As such, they do not form single crystals, and a definitive CIF file cannot be obtained. The structural description of the reduced films therefore necessarily relies on a combination of GI-XRD and GIWAXS data, from which the molecular packing motifs are inferred rather than fully resolved.

Third, for pristine neutral PDI films, the GI-XRD pattern exhibits three well-defined diffraction peaks at 12.0°, 25.1°, and 27.1°, which can be assigned to the (020), $(11\bar{2})$, and $(12\bar{2})$ lattice planes, respectively, based on the known single-crystal structure (Response Fig. 2a-d). When combined with the orientation information extracted from GIWAXS, these diffraction features allow us to construct a physically consistent schematic representation of the molecular packing in the neutral PDI films (Response Fig. 2e).

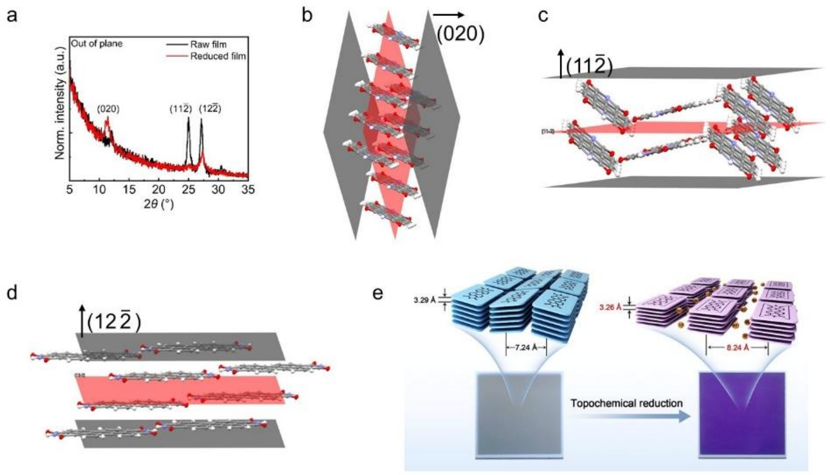

Response Fig. 2 a. Comparison of GI-XRD pattern between raw films and topochemical-reduced films. Molecular arrangements on crystal planes extracted from PDI single crystal and powder diffraction data are presented in b (020), c $(11\bar{2})$, d $(12\bar{2})$. e. Molecular stacking structure before and after reduction derived from diffraction/scattering data.

Upon topochemical reduction, the GI-XRD pattern of the PDI films shows clear changes: the diffraction peak corresponding to the $(11\bar{2})$ plane disappears, the (020) peak shifts to lower angles, and the $(12\bar{2})$ peak shifts to higher angles. These changes indicate an expansion of the lamellar spacing and a contraction of the π-π stacking distance, respectively. Importantly, no additional diffraction peaks emerge after reduction, suggesting that the films do not undergo a complete structural reconstruction into a new crystalline phase.

When these GI-XRD observations are considered together with the post-reduction GIWAXS orientation data, they impose strong constraints on the possible packing motifs in the reduced films. Within these constraints, it is reasonable to infer that the reduced films selectively retain molecular packing motifs associated with the (020) and $(12\bar{2})$ planes, while the packing mode related to the $(11\bar{2})$ plane is largely disrupted. On

this basis, we propose the schematic molecular packing model for the reduced films shown in manuscript (Response Fig. 2e), which should be understood as a diffraction-constrained structural representation rather than a fully resolved crystallographic structure.

Taken together, these clarifications highlight that the reduced films retain a partially preserved long-range registry while undergoing local structural rearrangements upon reduction. This behavior is consistent with the concept of topochemical reduction, which aims to modify the electronic state while largely preserving the pre-organized molecular framework. We have revised the manuscript accordingly to better define the scope and limitations of the structural interpretation.

We have revised the text to more clearly distinguish between structural features that are directly supported by experimental diffraction data and those that are inferred by comparison with pristine PDI.

Revised text: "The diffraction peak corresponding to the (11$\bar{2}$) plane disappears, the (020) peak shifts to lower angles and no additional diffraction peaks emerge after reduction, suggesting that the films do not undergo a complete structural reconstruction into a new crystalline phase."

5. Separately, the AFM data themselves raise additional concerns, as the authors describe a decrease in crystallinity and orientation after reduction. In light of this, it becomes even more important to provide direct structural evidence for the reduced material (e.g. powder XRD or single-crystal data on the reduced compound, or at least

a more rigorous analysis of the film GIWAXS/GI-XRD patterns) rather than relying on the pristine PDI structure as a template. Related to this, the structural model in Fig. 1e involving "insertion of proton" into the lattice appears highly speculative in the absence of direct proof.

Response: We thank you for raising this point and agree that our original wording may have led to ambiguity. We have revised the manuscript to clarify the distinct purposes of the AFM, GIWAXS, and GI-XRD analyses.

We emphasize that the AFM measurements were not intended to assess crystallinity or molecular orientation. Instead, AFM was used solely to examine surface morphology and roughness before and after topochemical reduction. As shown in Supplementary Fig. 5, the root-mean-square roughness increases only slightly (from 2.44 nm to 4.83 nm), indicating that the reduction process does not induce significant surface roughening or large-scale morphological disruption. We have clarified this point explicitly in the revised text.

The molecular orientation was quantitatively evaluated using GIWAXS data rather than AFM. Specifically, the orientation degree was calculated from the azimuthal intensity distribution at $q \approx 1.91$ Å^{-1} and 1.93 Å^{-1}, following the standard method described in the Supplementary Information and summarized in Supplementary Table 1. Upon reduction, the orientation degree decreases from 67.8% to 50.0%, indicating partial loss of preferential orientation rather than complete structural disruption. This quantitative distinction has now been emphasized in the revised manuscript.

Regarding crystallinity, we note that a rigorous numerical crystallinity index

cannot be extracted without reliable density values for both the crystalline and amorphous phases of PDI, which are not available for the reduced films. Therefore, we refrain from assigning absolute crystallinity values. Instead, the reduction in crystallinity is described qualitatively based on well-established diffraction signatures, including the weakening of GI-XRD peaks and the reduced intensity of scattering rings in GIWAXS patterns. We have revised the text to clearly state that this conclusion is qualitative in nature.

Concerning the nature of the counterions introduced during topochemical reduction, we acknowledge that direct identification of counterions in film systems is experimentally challenging. Our assignment of protons as the dominant counterions is therefore based on indirect but consistent evidence. Specifically, the lamellar spacing increases by approximately 1 Å after reduction, which is comparable to the contact diameter of a proton and significantly smaller than that of other possible cations such as ammonium (~2.8 Å). This inference is also consistent with previous literature on hydrazine-based reduction of diimide systems (*Nat. Commun.* **2024**, *15*, 5047). We have revised the manuscript to clarify that the identification of protons is an indirect inference rather than a direct measurement.

Revised text: "After 6 hours of topochemical reduction, the RMS roughness of the original flat raw films increased from 2.44 nm to 4.83 nm (Supplementary Fig. 5). The crystallinity and orientation of the film (extract from peak intensity in GI-XRD and azimuth integration in GIWAXS) were reduced (Fig. 1d and Supplementary Table 1)."

"The changes of lamellar packing distance read from the integral image of

GIWAXS (Fig. 1e) was determined to increase by 1 Å, possibly due to the insertion of counter cations (speculated to be protons, considering the diameters of other cations) generated during the reduction, causing lattice expansion."

6. Supplementary Fig. 12 shows an apparently enhanced orientation of the diffraction feature around 10° after reduction, but this is neither described nor interpreted in the main text or the Supplementary Information, and an explicit interpretation of this feature is clearly needed.

Response: We thank you for the careful and insightful observation of this diffraction feature. We agree that this point requires clarification, and we have revised the Supplementary Information to explicitly address the origin of this peak.

In the reduced NDI films, the diffraction peak appearing at 10.3° in the GI-XRD pattern, corresponding to the scattering ring observed at $q_z \approx 0.73$ Å^{-1} in the GIWAXS measurements, stands for (010) crystal plane (Response Fig. 3a). This feature is assigned to the lamellar stacking of NDI molecules, with an interlayer spacing of approximately 8.59 Å. A similar lamellar stacking motif is also present in the reduced PDI films.

The appearance of this lamellar diffraction indicates that, after reduction, a fraction of molecules adopt an edge-on orientation with respect to the substrate, while the remaining population retains a predominantly face-on character. We emphasize that this represents a coexistence of molecular orientations rather than a complete reorientation of the film.

To further understand this structural feature, we have subsequently investigated the mesoscopic domain-scale structure of reduced NDI films using resonant soft X-ray scattering (RSoXS). Under horizontally polarized synchrotron radiation, the scattering rings exhibit clear horizontal-vertical anisotropy, with the scattering intensity perpendicular to the electric field direction being significantly stronger than that parallel to it (Response Fig. 3b). The extracted anisotropy factor $A(E)$ is approximately 0.65, indicating that molecules at domain interfaces are predominantly face-on within the domains (Response Fig. 3c), which necessarily implies the presence of a non-negligible fraction of edge-on-oriented molecules relative to the substrate (*J. Phys.: Condens. Matter* **2021**, *33*, 313001; *Annu. Rev. Mater. Res.* **2025**, *55*, 1; *J. Phys. Chem. B* **2025**, *129*, 3529). This observation provides a consistent explanation for the emergence of the lamellar diffraction peak at 10.3° in GI-XRD and the corresponding $q_z \approx 0.73$ Å^{-1} feature in GIWAXS.

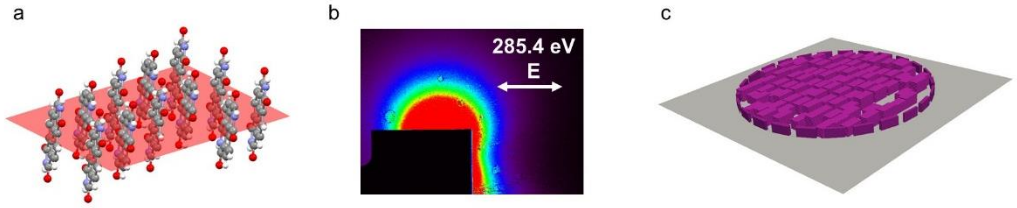

Response Fig. 3 a. Molecular arrangement on (010) crystal plane. b. Resonant soft X-ray scattering (RSoXS) pattern of ferromagnetic NDI films. c. Stacking model of phase domain scale with molecules on the surface face-on to the domain.

We note that a comprehensive discussion of the domain-scale morphology and its anisotropic scattering behavior would require a dedicated study and falls beyond the scope of the present work. We have therefore limited the discussion here to clarifying

the origin of the observed diffraction feature and have revised the Supplementary Information accordingly.

Revised text: "After topochemical reduction, the films show weaker crystallinity and orientation. The ($11\bar{2}$) peak shifted to smaller angle with a π-π distance of 3.26 Å, in agreement with the NDI radical powder reported before. The appearance of the signal at $q_z \approx 0.73$ Å^{-1} (10.3° in GI-XRD) can be assigned to the lamellar stacking of NDI molecules."

7. More fundamentally, it is not obvious that the procedure used to determine the film mass is sufficiently robust for a quantitative Ms value. Dissolving a reduced film and inferring its mass from the absorption of the resulting solution implicitly assumes complete and reproducible dissolution, a well-defined chemical composition (neutral/radical/dianion, counterions) that matches the calibrated molar absorptivity, and no material loss during transfer or handling. In a system where the active phase is a mixed-valence radical salt obtained by topochemical reduction, these assumptions are far from trivial. Any small underestimation of the mass (e.g. due to incomplete dissolution or incorrect extinction coefficients for the reduced species) will directly lead to an overestimation of Ms in emu g^{-1}.

Response: We deeply admire your rigorous scientific attitude. As you pointed out, accurate determination of the film mass is critical for evaluating the saturation magnetization. The details on the determination of the mass were not described with sufficient clarity in the original manuscript, and we therefore provide clarification and

corresponding revisions here.

In your comment, you raised two main concerns: whether the PDI in the film can be completely dissolved, and whether the dissolved PDI exists in a mixed-valence state. These are indeed critical issues in determining the film mass using spectroscopic methods. To address these two points, we employed an excess amount of hydrazine hydrate during the film dissolution to ensure that the film was fully transferred into solution. Meanwhile, the UV-vis spectrum of the resulting solution (Response Fig. 4a) showed clear PDI dianion absorption without peaks corresponding to the neutral state or the singly reduced radical species (*Chem. Res. Chin. Univ.* **2023**, *39*, 187-191; *Natl. Sci. Rev.* **2024**, *11*, nwae087.), indicating that the dissolved PDI was completely reduced to the dianions. Furthermore, to minimize errors associated with material loss during transfer or handling, a standard calibration curve was established using absorbance data (Response Fig. 4b). This allows the use of literature-reported extinction coefficients for the corresponding dianion species, rather than those of a mixed-valence solid-state system. Repeated dissolution and absorbance measurements yielded consistent results, supporting the reproducibility of this approach.

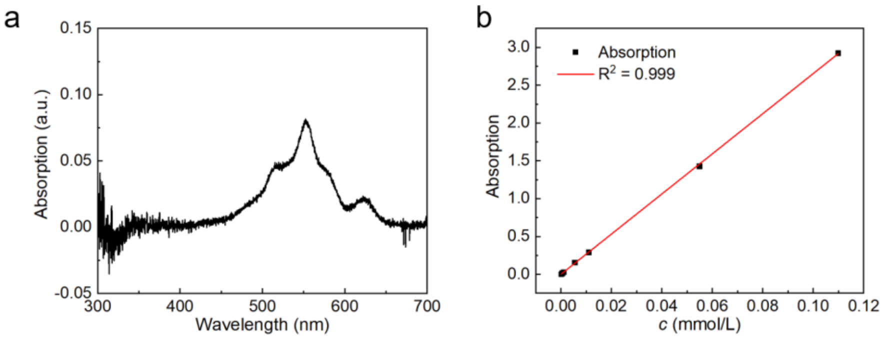

Response Fig. 4 a UV-vis spectra of film disolved in an excess amount of hydrazine hydrate. **b** Standard curve of absorption-weight.

Generally, determining film mass from solution absorbance is a widely accepted and well-established approach in analytical chemistry, particularly for organic films where direct weighing is impractical. This method has been extensively used for quantitative analysis of conjugated organic molecules and films based on the Lambert-Beer law, provided that complete dissolution and appropriate calibration are ensured (*Powder Technol*. **2024**, *446*, 120087; *J. Electron. Mater*. **2024**, *53*, 6012; *Dalton Trans*. **2025**, *54*, 16268).

We have revised the manuscript to clarify the methodology and explicitly address the concerns.

Revised text: "We determined film mass from solution absorbance.[1-5] A series of concentrations of PDI solutions were prepared. Considering the final concentrations below 10^{-4} mol/L, the PDI species were fully converted into dianion state. By fitting the standard curve, we calculated its slope to be 26.54 and intercept to be 1.6508×10^{-4}. After fully dissolving the film in hydrazine hydrate, the absorbance of the solution at 553 nm was 0.08097, which was incorporated into the curve to obtain a film weight of 5.95 μg."

****

Once again, we acknowledge the editor's and the reviewer's comments and constructive suggestions very much, which are quite valuable in improving the quality of our original manuscript.

Responses to the reviewers' comments

We thank the reviewers for their positive evaluation of our manuscript.

As requested by the editor, we have revised the manuscript to address the editorial requests listed in the Author Checklist. No further scientific comments from the reviewers were raised in this round.

We hope that the revised manuscript meets the requirements for publication in Nature Communications.
